# Adaptive feature fusion based semi-supervised federated learning system for ECG arrhythmia detection

**DOI:** 10.3389/frai.2026.1913613

**Published:** 2026-09-09

**Authors:** Silla Ann Regi, Jagadeesan Srinivasan, Thanapal Pandi

**Affiliations:** 1 Department of Computer Applications, School of Computer Science Engineering and Information Systems, Vellore Institute of Technology, Vellore, Tamil Nadu, India; 2 Department of Software and Systems Engineering, School of Computer Science Engineering and Information Systems, Vellore Institute of Technology, Vellore, Tamil Nadu, India

**Keywords:** adaptive feature fusion, arrhythmia detection, ECG signals, federated learning, healthcare AI, machine learning, pseudo labeling, semi-supervised learning

## Abstract

Automatic cardiac arrhythmia detection using electrocardiogram (ECG) signals is essential for early diagnosis of cardiovascular conditions. Most of the previously existing systems for arrhythmia detection depend on fully supervised learning approaches. In addition to that, since the ECG patient data is centrally stored, it can raise concerns like privacy and security in healthcare environments. To address these issues, this work explores a semi-supervised federated learning framework that detects arrhythmia using ECG signals. The MIT-BIH Arrhythmia dataset is used from which the heartbeat segments are extracted and relevant features are obtained. The framework incorporates the use of multi-level feature extraction, adaptive feature fusion and pseudo-labeling to utilize both labeled and unlabeled data efficiently. In order to improve the performance of the model, a weighted federated aggregation approach is used where the contribution of each of the clients is considered during the global model updates. Implementation and evaluation of multiple machine learning and deep learning models including Random Forest, Support Vector Machine, K-Nearest Neighbor, AdaBoost, Gradient Boosting and Multi-Layer Perceptron are performed within the proposed framework. This proposed approach achieves an accuracy of 96.81% in semi-supervised federated settings and has an AUC value of 0.9957 that shows high classification reliability. The system helps improve the model learning with limited number of labeled data at the same time ensuring privacy preserving distributed training. The model maintains a competitive performance under federated settings as compared to the centralized training. Among the evaluated models, ensemble and neural network based approaches show superior performance. The framework demonstrates a stable performance across federated training rounds. Overall, the results obtained show the effectiveness of the proposed framework for arrhythmia detection in a simulated federated setting.

## 1 Introduction

Over the recent years there has been an integration of artificial intelligence into the healthcare environments which has brought a very drastic change or transformation in the way in which data in medical field is analysed, interpreted and utilized for diagnostic uses. Machine learning and deep learning branches have proved to be great tools for identifying complex patterns within large scale biomedical data. One such scenario where biomedical data is being used include the automated detection of cardiac arrhythmia by using electrocardiogram (ECG) signals. Out of the many causes leading to mortality around the globe, cardiovascular diseases still remain as one of the main reasons for mortality. Arrhythmia is one among these conditions where there are irregularities in the heartbeat rhythm. Detection of arrhythmia consumed much amount of time, was resource intensive and also there existed chances of human errors when it comes to large scale scenarios. The sudden and large increase in the generation of continuous data due to the increase seen in the adoption of wearable health monitoring devices and digital healthcare platforms has led to an urgent requirement for automated, scalable and efficient systems that have the capability of processing and analysing medical data in real time. This shows the significance of machine learning and deep learning techniques in this domain area where these models could actually automatically classify ECG signals into normal and arrhythmic patterns. Most of the approaches that exist in current scenario for ECG-based arrhythmia detection depend mostly on fully supervised learning techniques which needs huge number of data that is labeled accurately. This requires expert annotation and high costs associated with it. Also, there is a requirement for aggregation of patient data gathered from many sources into one location for using traditional centralized machine learning frameworks. This raises a serious concern in areas like data privacy, security and regulatory compliance. These above listed challenges are addressed using some of the emerging paradigms like semi-supervised learning and federated learning. Models are allowed to use both labeled and unlabeled data in semi-supervised learning. This helps in reducing the dependency on extensive annotated datasets. Decentralized model training can be done using federated learning. Here data will be remaining localized to its source, it could be hospitals or any wearable devices and only the updates from the model will be shared. This helps to ensure preservation of privacy. Using these advancements, this project aims to build an advanced framework for ECG-based arrhythmia detection that would be integrating both semi-supervised learning and federated learning along with using adaptive feature fusion mechanisms. The system is developed in a particular manner that it efficiently uses heterogenous ECG features. This includes raw signal characteristics, statistical attributes and wavelet-based frequency features which helps improve classification performance. Techniques like adaptive pseudo labeling and weighted federated aggregation is also used to evaluate the proposed framework under simulated distributed settings and heterogeneous data distributions. The proposed system focusses on bridging the existing gap that persists between accuracy, data efficiency and privacy-aware distribution learning in healthcare AI systems. Since the present study focusses on a single public dataset and simulated federated clients, the proposed system can be considered as a preliminary approach which needs to be validated on diverse datasets and real-world clinical environments before it is deployed.

The contributions of this project include the following:

- Developing a hybrid learning framework for detecting arrhythmia.
- Integrating semi-supervised learning for label efficient training.
- Implementing a federated learning framework that can reduce the need for direct sharing of raw ECG data.
- Extracting multi-level features from the ECG signals.
- Incorporating the technique of adaptive feature fusion.
- Using an adaptive confidence-based pseudo labeling.
- Applying the strategy of weighted federated aggregation.
- Implementing and comparing among multiple machine learning models.
- Evaluating the performance using standard metrics.

## 2 Related work

The literature review on arrhythmia detection has led to the understanding that there was a rapid advancement of both machine learning and deep learning techniques to significantly enhance the automatic detection of arrhythmia condition using ECG signals as input data. There have been various research studies that has used various approaches that include machine learning models, deep learning architectures, hybrid frameworks and even feature fusion techniques. Even though these models are existing, there are limitations that exist such as more dependence on labeled data, high computational complexity, lack of generalization and concerns that are associated with data privacy. The review that follows below shows the prior works on arrhythmia detection focusing on the methods developed, frameworks, and gaps identified.

Major advancements have been identified in the field of arrhythmia detection specifically ECG-based by following the process of combining machine learning and deep learning methods. Combining handcrafted feature extraction with traditional classifiers was the main focus applied in the early approaches. For instance, an efficient classification model that was based on Chi-square distance integrated along with Particle Swarm Optimization (PSO) was suggested by Al-Shammary et al. (2024) for feature extraction. The technique was successful in achieving an accuracy which is close to 98% approx. Which was ideal in showing that for improving classification performance, optimized feature selection was important. But the suggested approach depended highly on features that were engineered manually and also lacked the adaptability to diverse and large-scale datasets. The limitations that were identified shows that there is a need for feature learning that is automated and also adaptive feature fusion. Hybrid architectures were introduced once after the evolution of deep learning for capturing characteristics like spatial and temporal characteristics of ECG signals. An explainable attention-based CNN-GRU model was developed by Ba Mahel et al. (2025). The model had the ability to classify multiple types of arrhythmias and achieved an accuracy which is close to 99.16%. when attention mechanisms were integrated, it improved the interpretability which is again crucial in healthcare applications. The model as suggested by the author needed large labeled datasets and also significant computational resources. This shows the need for using a semi-supervised learning approach to use unlabeled data efficiently. There were also studies that focused on using transformer based architectures for analysing the ECG signals. As proposed by Hu et al. (2022), a transformer-based model called ECG-DETR was developed that performed an end-to-end detection of arrhythmia without the need for any explicit segmentation of the heartbeats. This helped the model to achieve an accuracy exceeding 99%. Even though this model was successful in simplifying the detection pipeline, it required large datasets and high computational power. This shows the significance of developing a scalable and computationally efficient system. Classification performance was improved once feature fusion emerged. As proposed by Din et al. (2024), an ensemble learning framework that used various models like CNN, LSTM and Transformer models achieved an accuracy which is close to 99.56% after the models captured spatial, temporal and long-range dependencies. Model complexity is a matter of concern even though fusion approach enhances the performance. This shows the need for an adaptive feature fusion-based technique for balancing between performance and computational efficiency.

There was also a similar study performed by Duranay et al. (2025) who performed an exploration on the area of Vision Transformers (ViT) for classification of ECG signals. Here the system proposed achieved an accuracy which is close to 96.79% and also showed improved interpretability. There could be chances of information loss and increased processing overhead when ECG signals are transformed into images. This shows the need for directly making use of signal based and extracted features. Multiple architectures have been integrated to form hybrid deep learning architectures. A CNN-GNN-BiLSTM framework was developed by Mahajan and Kaul (2025) to capture spatial, relational and temporal dependencies. This helped achieve an accuracy of 99.89%. these kinds of frameworks are highly effective but at the same time led to computational expense and difficulty in real time deployment. This shows the need for maintaining a balance between accuracy and efficiency. There are also lightweight models that were introduced to cope up with these computational challenges. A CNN-LSTM based model was proposed by Alamatsaz et al. (2024) that showed an accuracy of 98.24% and also maintained a reduced complexity. Even though these kinds of models show efficiency, they fail in capturing complex ECG patterns. This shows the need for using multi-feature extraction and adaptive learning techniques for enhancing the robustness. Hardware oriented implementations have also been carried out apart from the software-based approaches. A CNN based arrhythmia detection system was developed by Mangaraj et al. (2025) and was optimized for FPGA deployment with an accuracy of 97.79%. Although this development was suitable for real time applications, model flexibility was limited due to hardware constraints. This shows the need for a scalable work that can be used for future deployment. Dhyani et al. (2023) used a 3D wavelet transform along with SVM for classifying arrhythmia. This achieved an accuracy of 99.02%. in certain scenarios like these, the traditional approaches are still relevant. But such kinds of features are dependent on handcrafted features and also do not have the automatic feature learning capabilities. This shows the need for an adaptive feature fusion technique. A CNN based arrhythmia detection model was developed by Fahad et al. (2024) and achieved an accuracy of 98.3%. Even though model was efficient, it did not use any kind of advanced learning paradigms like semi-supervised learning or federated learning. As per a comprehensive review that was conducted over 200 studies by Ayyub et al. (2025), it was found that deep learning models often outperform traditional approaches in analysis of ECG signals. The research also denoted that there is a lack of standardization, data variability and privacy concerns which can be considered as critical challenges. This shows the need for using a federated learning approach in the current systems.

A hybrid Spindle Autoencoder-CNN model was proposed by Akkuş et al. (2025) that was successful in achieving an accuracy of 98.78%. Even though the model could perform extraction of deep features effectively, it still needed the validation on larger datasets. This shows the need for scalable and generalizable systems. As developed by Choudhury et al. (2023), a deep learning model could be optimized by using an Exponential Political Optimizer. This helped to achieve a moderate accuracy and improved optimization. But this kind of approach led to the increase in need for computational overhead and shows the need for efficient optimization strategies. A CNN based model using Wavelet Scattering Transform was developed by Nahak and Saha (2025) for an interpretable ECG classification. Even though the interpretability was improved, it failed in maintaining a good accuracy across diverse datasets. A denoising and deep ensemble learning architecture was proposed by Lamba and Diwakar (2026) making up an accuracy of 99.9%. Even though it shows high performance, the framework increases the system complexity and computational cost because of multiple models. A comparative study was conducted by Nugraha et al. (2024) comparing various models like CNN, KNN and SVM and CNN demonstrated superior performance. This shows how effective deep learning approaches can be effective in ECG classifications. A machine learning based system that used ECG sensors was implemented by Tabhane et al. (2023) and resulted that random forest achieved the best performance. But the system still lacked the use of advanced methods that can help improve scalability and adaptability. A fetal arrhythmia detection was proposed by Nakatani et al. (2022) that helped to achieve good accuracy but still had limitations like signal noise and variability. Multiple machine learning models were explored by Yu (2023) which included clustering and neural networks as well. This got moderate improvements in classification accuracy but still showed performance that was lower than other deep learning models. Grad-CAM based dynamic weighted feature fusion approach was introduced by Khatar et al. (2025) and showed high accuracy along with improved interpretability. But here the complexity of the model limited the real time applicability. This shows the need for a simpler and at the same time an effective adaptive feature fusion strategy. On the whole, there are existing studies and researches that show a significant progress in detection of ECG-based arrhythmia by using techniques like machine learning and deep learning. But there are still limitations that persist like lack of privacy preserving techniques, dependency on large labeled datasets and also increase in the complexity of the model. These limitations shows that there is a need for an integrated, efficient and scalable framework. A tabularized form of the above discussed studies has been shown below in Table 1.

**Table 1:** Comparative review of machine learning and deep learning techniques for ECG-based arrhythmia detection.

S. No.	Paper	Dataset used	Objective	Result	Future work
1.	Al-Shammary et al. (2024)	MIT-BIH Arrhythmia Dataset	The system proposed does arrhythmia detection using ECG records and this is useful for both academic research and medical use. It makes use of PSO full formed as Particle Swarm Optimization and also Chi-square distance that helps in measuring the performance and also optimizing it.	Accuracy rate close to 98% with PSO was obtained, which is higher than 89% that was achieved with just chi square.	Limited dataset and generalization, Lack of robustness analysis under real-world noise, Computational efficiency and real-time deployment
2.	Ba Mahel et al. (2025)	MIT-BIH Arrhythmia Dataset, PTB Dataset	The hybrid model proposed in this paper uses XABH-CNN-GRU based hybrid model which has the capability to find out five types of heartbeats which included types like atrial premature (A), ventricular premature (V) etc.	A hybrid model that makes use of attention mechanism, by using electrocardiogram (ECG) data. Incorporated an explainability feature into the model. Accuracy: 99.16%	Insufficient representation of arrhythmia classes, Limited dataset diversity and clinical coverage, Need for broader benchmarking and optimization
3.	Ayyub et al. (2025)	MIT-BIH Arrhythmia Dataset + PTB + CPSC 2018 + PhysioNet Dataset	Systematically review and critically analyse AI-based methods (Machine Learning and Deep Learning) used for detection of arrhythmia using ECG signals.	Deep learning models outperform traditional ML, MIT-BIH is the most dominant dataset, there is a rapid growth in AI-based ECG research	Focus on standardizing ECG data, pre-processing, and evaluation methods, and validating AI models on larger, diverse real-world clinical datasets. Developing explainable, robust, and computationally efficient models
4.	Hu et al. (2022)	MIT-BIH Arrhythmia Dataset, MIT-BIH AFIB Database, PTB-XL Dataset	This study uses a deep learning model called ECG-DETR that uses a transformer framework. This analyses the continuous single-lead ECG data for finding out arrhythmia.	It reports 99.49% accuracy (4-class task), 99.12% accuracy (8-class task), and 99.23% accuracy for atrial fibrillation detection	Various other methods can also be tried to perform arrhythmia detection and also use the system developed for detecting other kinds of signal detection issues.
5.	Akkuş et al. (2025)	MIT-BIH Arrhythmia Dataset	Develop a hybrid deep learning model that uses a Modified Spindle Autoencoder (MSCAE) and CNN for accurate ECG-based arrhythmia classification.	Got a maximum classification accuracy which is close to 98.78% by using the MIT-BIH Arrhythmia dataset	Improve performance for complex and minority arrhythmia classes (especially S and V beats), Validate the model on another bigger, more different real-world clinical ECG datasets
6.	Din et al. (2024)	MIT-BIH Arrhythmia Dataset	Develop an arrhythmia detection system that is based on ECG signals using ensemble learning with deep feature fusion. Combine CNN, LSTM, and Transformer models to capture spatial, temporal, and long-range dependency features from ECG signals.	99.56% accuracy and 99.34% F-score	Extend the model from binary classification to multi-class arrhythmia detection.
7.	Duranay et al. (2025)	Multi-lead ECG Dataset	Evaluate the effectiveness of Vision Transformer (ViT) models for multi-channel ECG arrhythmia detection using image-based representations, to convert 12-lead ECG signals into high-resolution images, applying multiple Explainable AI (CAM)	ViT-B/384 model achieved the best performance, with accuracy close to 96.79% and f1-score close to 96.78% for four-class arrhythmia classification	Validate the approach on larger, multi-source real-world ECG datasets beyond a single public database,
8.	Mahajan and Kaul (2025)	MIT-BIH Arrhythmia Dataset + PTB Dataset + Chapman-Shaoxing Dataset	Deep learning framework that is graph enhanced (CNN–GNN–BiLSTM) for accurate ECG arrhythmia detection, jointly model spatial features (CNN), inter-beat relationships (GAT), and temporal dependencies (BiLSTM)	Achieved 96.3% overall accuracy across combined datasets and 99.89% accuracy on the MIT-BIH dataset.	Validate performance on anther vast and more different or unique real-world datasets related to healthcare, Optimize the framework for real-time and wearable ECG monitoring applications.
9.	Choudhury et al. (2023)	MIT-BIH Arrhythmia Dataset + MIT-BIH Normal Sinus Rhythm Database	Develop an EPO (Exponential Political Optimizer) – that was trained Deep Quantum Neural Network (QNN) for automatic ECG-based detection of arrhythmia.	91.4% accuracy, 92% sensitivity, and 91.7% specificity on the MIT-BIH datasets	Enhance or increase accuracy of detection by identifying more powerful and discriminative characteristics.
10.	Alamatsaz et al. (2024)	MIT-BIH Arrhythmia Dataset + Long-Term AF Database	Develop a lightweight CNN–LSTM deep learning model for accurate arrhythmia detection that is ECG-based, classify 8 different arrhythmias along with normal rhythm using raw ECG signals, improve model interpretability by integrating SHAP-based explainable AI.	Average classification accuracy of 98.24%, showed high sensitivity and specificity (>90%)	Improve classification performance for certain difficult arrhythmia classes, incorporate additional ECG features
11.	Nahak and Saha (2025)	MIT-BIH Arrhythmia Dataset	Analyse and compare CNN and Wavelet Scattering Transform (WST) for arrhythmia classification using beat-level ECG.	1D-CNN achieved close to 98.74% (intra-patient) and 94.50% (inter-patient) accuracy, while the WST achieved 97.73 and 95.59%	Improve classification of minority and difficult arrhythmia classes
12.	Mangaraj et al. (2025)	MIT-BIH Arrhythmia Dataset + INCART 12-Lead Dataset	Design a dual-branch, hardware-aware 2D-CNN architecture FPGA-based arrhythmia detection system using quantized ECG beat images	Achieved classification accuracy close to 97.79% by using dataset from the MIT-BIH Arrhythmia Database.	Extend the system to support more arrhythmia classes and multi-lead ECG signals, Validate and deploy the system on more vast and more different real-world ECG datasets.
13.	Lamba and Diwakar (2026)	MIT-BIH Arrhythmia Dataset + MIT-BIH Normal Sinus Rhythm Database + St. Petersburg INCART Database	Develop an integrated ECG arrhythmia detection framework that jointly addresses signal denoising and classification and a novel DWT with Fractional Total Variation (DWTFrTV) method for effective ECG noise removal.	Achieved very high accuracy: 99.9% (training) and 99.7% (testing) by using dataset from the MIT-BIH Arrhythmia Database.	Extend the architecture to handle more arrhythmia classes and multi-class classification instead of binary classification.
14.	Dhyani et al. (2023)	CPSC 2018 Dataset	Develop an arrhythmia detection system that is ECG-based that uses 3D Discrete Wavelet Transform (DWT) for denoising and extracting features and classify ECG signals to either normal and abnormal rhythms using a Support Vector Machine (SVM).	High classification accuracy of up to 99.02%	Extend the binary classification system to multi-class arrhythmia classification for clinical decision support.
15.	Khatar et al. (2025)	MIT-BIH Arrhythmia Dataset	Introduce a Grad-CAM–guided dynamic weighted fusion of Gramian Angular Field (GAF) representations for better feature relevance and design a hybrid deep learning architecture (Parallel-Residual CNN + Bi-LSTM) for robust arrhythmia classification	Achieved accuracy close to 98%, sensitivity close to 99%, and F1-score close to 98% on the MIT-BIH Arrhythmia Database.	Extend the proposed architecture to other cardiac abnormalities and broader clinical datasets. Further refine the fusion strategy to improve performance on ECG leads with modest gains (e.g., V3 and V4).
16.	Nugraha et al. (2024)	MIT-BIH Malignant Ventricular Ectopy Database	Comparative analysis of machine learning and deep learning methods (CNN, KNN, SVM) and classify ECG fragments into six risk-based arrhythmia categories using spectral features	CNN achieved the best performance with accuracy close to 89%, precision close to 89%, recall, and F1-score close to 89% in multi-class classification	Improve performance by using vast and more different ECG datasets instead of relying on a single database. Explore advanced deep learning architectures, ensemble models, and better hyperparameter tuning, especially for SVM.
17.	Tabhane et al. (2023)	Custom ECG Dataset collected using AD8232 ECG Sensor and Arduino Uno	Design a cardiac arrhythmia detection system using the AD8232 ECG sensor and machine learning algorithms and compare machine learning models (Random Forest, Logistic Regression, Gaussian NB, Decision Tree)	Random Forest achieved the most performance with an accuracy close to 92.59%	Improve performance by using vast and more different ECG datasets. Explore additional features and advanced machine learning/deep learning models.
18.	Fahad et al. (2024)	MIT-BIH Arrhythmia Dataset	Creating a deep learning model that is CNN-based for automatic detection of cardiac arrhythmia using ECG signals.	Achieved a testing accuracy close to 98.3% and a validation accuracy close to 83.78%.	Improve validation performance by using larger and more diverse ECG datasets. Explore advanced CNN architectures or hybrid deep learning models to enhance generalization.
19.	Nakatani et al. (2022)	PhysioNet Fetal ECG Arrhythmia Database	Develop a deep learning–based fetal arrhythmia detection method using raw Fetal ECG (FECG) waveforms	Proposed CNN-based method achieved up to 96.2% accuracy, with 100% recall and 100% specificity for fetal arrhythmia detection.	Make the approach more practical for real-world clinical deployment by reducing parameter sensitivity
20.	Yu (2023)	Custom ECG Dataset	Design an automatic arrhythmia classification and detection system using multiple machine learning algorithms to reduce manual ECG analysis errors.	Clustering (k-means) showed low classification accuracy. ANN model achieved 83.93% accuracy. GA-optimized ANN improved accuracy to 87.95%	Integrate the trained model into real-time ECG monitoring and detection devices for clinical use, optimize feature selection and learning algorithms to improve classification accuracy for complex arrhythmia types, extend the approach to larger and more diverse ECG datasets

## 3 Threats to validity

Several threats to validity must be considered though promising results were achieved using the proposed semi-supervised federated learning framework for detection of arrhythmia. The proposed system’s performance can be influenced by the choice of pre-processing techniques, feature extraction methods and model configurations. The dependencies on parameter selection like confidence thresholds are introduced by using adaptive feature fusion and pseudo labeling techniques. This can affect the model’s behavior. Also, synthetic patterns that may not fully represent the real-world ECG variations may be introduced by handling the class imbalance using SMOTE. The MIT-BIH Arrhythmia dataset is used which is one of the most widely used dataset. This dataset may not fully capture the diversity of the real-world clinical data. There can be variations in the ECG signals due to the differences in demography, sensor types and acquisition conditions. This may impact the generalizability of the model when it is deployed in various healthcare systems. Their study focuses in binary classifications (normal vs. arrhythmia) which may limit the applicability to the multi-class arrhythmia detection scenarios. Basic standard performance metrics like accuracy, precision, F1-score and recall are n = being used to evaluate the model but these may not help in fully capturing the clinical relevance or the interpretability required in real-world healthcare applications. Also, pseudo-labeling depends on model confidence and this may propagate the errors if incorrect labels are assigned while training. The conclusions are drawn by using experimental results that were obtained under controlled settings like simulated federated environments. The reliability of conclusions regarding scalability and communication efficiency may be limited due to the absence of real-world distributed environment.

Although MIT-BIH Arrhythmia Database, a widely recognized and used benchmark for ECG arrhythmia research was used to evaluate the proposed framework, its architecture is not limited to a single dataset. The pre-processing pipeline, adaptive feature fusion strategy and semi-supervised federated learning framework can be adapted to various other ECG datasets given that appropriate pre-processing and annotation mapping is done. If the proposed approach is evaluated on additional publicly available and real-world clinical datasets, it would help in further validating the generalizability and robustness across diverse patient populations and recording conditions.

Even though the proposed system shows promising performance, there could be several practical challenges that would arise when the system is deployed in real time. Training time could increase, especially in the large scale healthcare networks when there is communication overhead between distributed clients and central server. Also, the variations in computational resources, data quality and patient demographics across the various participating institutions can lead to client heterogeneity and non-IID data distributions. This may sometimes affect the model’s convergence and performance.

Overall, there is a need for further validation on diverse datasets and real-world federated deployments to strengthen the robustness and applicability of the findings even though the proposed framework shows strong performance and promising potential.

## 4 Research gap

Centralized supervised learning frameworks are being used in most of the current arrhythmia detection systems. There are two main limitations associated with this. One is the need for large amounts of labeled datasets which is difficult to get because annotation of medical data will require expert intervention and significant manual effort. And secondly, if the data collection is centralized it can lead to another set of serious concerns that include data privacy, security and regulatory compliance. Effective utilization of heterogenous nature of ECG data is one other area where many of the existing models fail. Some of the other limitations include the lacking of robust learning mechanism that has the ability to operate across various multiple sources. Data is mostly decentralized inherently, presiding over different clinics, hospitals or devices when it comes to real-world healthcare scenarios. The existing systems do not have the ability to or are not designed in a particular way that it can learn collaboratively without compromising the concern of data privacy in such environments. Even though techniques like semi-supervised learning and federated learning are used to address some of these issues, these techniques were performed independently without any integration and advanced feature representation strategies. This leads to limitation in cases of performance improvements and also reduced adaptability in complex healthcare environments.

The following are the research gaps that were found:

### 4.1 Dependency on large labeled datasets

Large labeled datasets are difficult to obtain in medical scenarios and also expensive. But this is what most of the machine learning models require. This leads to limitations in scalability and practical usability.

### 4.2 Unlabeled data remains underutilized

Most of the ECG data that exists are left unlabeled and there are no many approaches that make use of this in an effective way. Semi-supervised learning can be used to thereby increase the model’s efficiency.

### 4.3 Lack of privacy preserving mechanisms

If the approaches used for training is centralized, it requires sharing of data. This can lead to serious privacy issues as well as security concerns. There are only a few studies that have implemented decentralized learning.

### 4.4 Inefficient representation of feature

Many models that used for arrhythmia detection uses only single type features or simple combinations. These do not consider feature importance. This may lead to suboptimal classification performance.

### 4.5 High computational complexity

When transformers and ensembles are used, it need higher computational resources as these are advanced models. This makes it not usable in real time and resource constrained environments.

### 4.6 Limited generalization across datasets

Most of the models that were trained used specific datasets. This led to fail in generalizing diverse real-world data. This can affect the reliability and robustness.

### 4.7 No integrated learning frameworks found

Mostly individual techniques are used instead of combining multiple approaches. It can affect the overall effectiveness of the system.

### 4.8 Lesser focus on real-world deployment

Most of the testing of the systems are done in controlled environments only and end up ignoring the real-world challenges. This can limit the aspect of practical applicability.

### 4.9 Lack of adaptive learning mechanisms

Fixed thresholds and static parameters are used often by the existing models. This may lead to reduction in flexibility when varying data distributions are handled.

### 4.10 Interpretability and reliability found to be insufficient

Most of the models used actually act as black box systems which can lead to trust issues especially for the medical professionals. So, interpretability is a key issue.

So, there is a requirement for a unified system that can make sure that data efficiency, privacy, adaptive feature handling and scalability are maintained.

## 5 Methodology

The work proposes a framework for efficient arrhythmia detection using ECG signals while reducing the need for direct sharing of raw ECG data in distributed training. The overall flow of the framework includes various stages including the dataset preparation, pre-processing, feature extraction, feature fusion, semi-supervised learning, federated learning, training the models and its evaluation. The dataset used for this study is taken from the MIT-BIH Arrhythmia Database that contains data in the form of annotated ECG recordings. From these ECG recordings, a fixed window size is used to do extraction of heartbeat segments. The beats that are extracted are labeled as either of the two classes: normal and arrhythmic. Minority Oversampling Technique (SMOTE) is used to make sure that classes are properly balanced and thereby helps in improving the training and performance of the model. Pre-processing of the ECG signals is performed to remove noise and inconsistencies that exist. It involves steps like normalization and segmentation of signals. The ECG signals are segmented into fixed length samples. Once segmented, the meaningful representations are obtained from the signals by performing feature extraction. This includes the raw ECG signal features, statistical features and wavelet-based features. Both time-domain and frequency-domain characteristics of the ECG signals can be derived from the features. An adaptive feature fusion mechanism is used to combine the extracted features based on their variance-derived weights. This provides different relative contributions to the feature groups while producing a fused representation for subsequent classification. The dataset is split in the ratio 80:20 to form training set and testing set. Data within the training set is then again divided into labeled and unlabeled for creating the semi-supervised setup. Then the model assigns the labels to these unlabeled data depending upon the prediction confidence. An adaptive confidence-based pseudo labeling strategy is being used here. High confidence samples are selected using a dynamic threshold. These samples are used to improve the learning during the training process. Federated learning approach is used to simulate multiple virtual clients. The training data is distributed across these clients where each client actually simulates any local data source. Local model training will be done within each client using its local data and does not share the raw ECG signals. A weighted federated averaging technique is used to aggregate the models that were trained locally. Here the size of the data of each client is considered to adjust the contributions of each client. A global model is then obtained by repeating this same process for multiple communication rounds.

**Figure 1:**
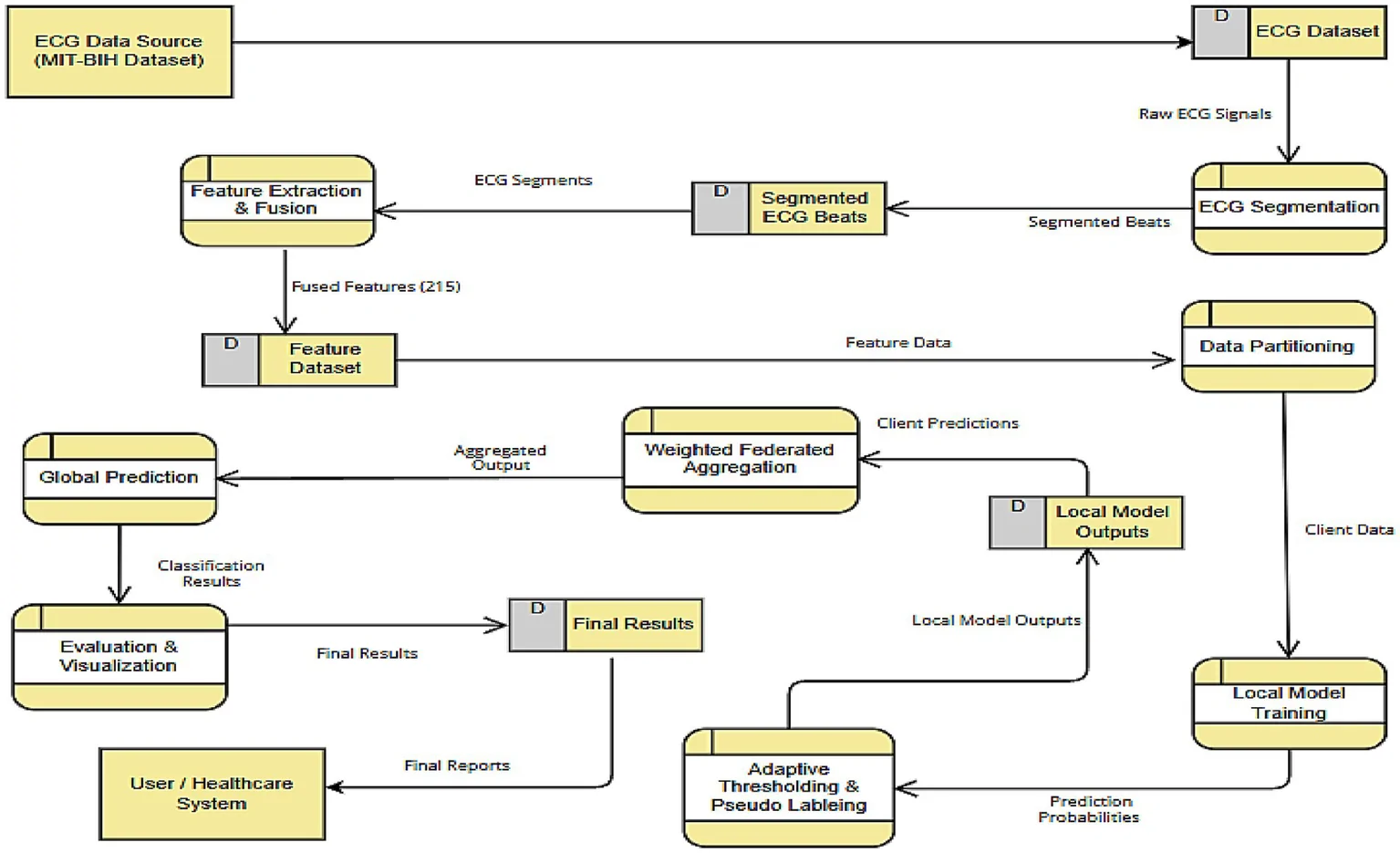
Logical flow diagram for proposed system.

Multiple machine learning models like Random Forest, Support Vector Machine, K-Nearest Neighbors, AdaBoost, Gradient Boosting and Multi-Layer Perceptron are being implemented within the framework. The model that is primarily used for core semi-supervised federated learning is Random Forest. The other mentioned models are being used for comparative analysis. All the above-mentioned models are being trained and evaluated using various configurations like centralized, federated and semi-supervised settings. The performance evaluation of the proposed system is carried out using various standard metrics. This includes accuracy, precision, recall, f1-score, ROC and AUC. Confusion matrix, ROC curves, feature importance and interpretability techniques are the other metrics that are used for analysis and understanding the model’s behavior. The entire implementation of the proposed framework is done in Python language and in Google Colab environment. Figure 1 clearly shows the logical flow of data in the system, all of this as stated earlier in the system.

## 6 Proposed system

The proposed system focusses on developing an advanced and integrated system framework for ECG-based detection of arrhythmia. It makes use of multiple machine learning techniques along with modern learning paradigms so that the limitations of the existing systems can be addressed. The system is made in a way that it can improve the accuracy while classification, use the available data in an efficient manner and also reduce the need for direct sharing of raw ECG data in distributed training environments. The proposed system uses a semi-supervised learning framework in which the models are made to learn from both kinds of ECG data, i.e., labeled and unlabeled. This helps to improve the overall learning efficiency and also bring a reduction in the dependency on large datasets that are annotated. The unlabeled data is labeled using pseudo labeling strategy which helps to enhance model training by assigning high confidence predictions on unlabeled data. Pseudo label selection is implemented by using an adaptive confidence-based threshold mechanism. This is different from the static or fixed thresholds in a way that this uses a dynamic approach to help in improving the reliability of semi-supervised learning. The data privacy concerns associated with the existing systems are addressed using a federated learning mechanism. Using this approach, multiple clients are made to train the models locally. These clients are used to represent different data sources like any devices or hospitals. So, here the raw ECG data is not being shared. The communication and aggregation of model parameters take place. This helps in keeping the raw ECG data localized to the respective client during the simulated federated training process and also may reduce the risks in exposure of data associated with centralized data sharing. However, federated learning alone does not guarantee complete privacy, since model parameters or updates may potentially reveal sensitive information. To improve feature representation, an adaptive feature fusion technique is also introduced. Features like raw ECG signals, statistical features and wavelet-based features are extracted and combined using a weighted approach. This will be assigning the importance depending upon their contribution. This helps to increase the capability of the model to identify the complex patterns in the ECG signals. The proposed framework uses a number of machine learning models like Random Forest, SVM, KNN, Gradient Boosting, AdaBoost and Multi-Layer Perceptron (MLP) for classification. These models are used for proper evaluation and comparison and thereby finding out the most effective approach that can be used for detection of arrhythmia. There is also an application of a weighted federated aggregation strategy in which each of the client contributions are being adjusted depending upon the data size. This makes sure that the updates from the model are fair and effective even across distributed environments. On the whole, the system aims to provide a data-efficient distributed solution for detecting arrhythmia. The integration of semi-supervised learning, federated learning and adaptive feature fusion enables evaluation of the framework under simulated distributed settings. The present study does not establish real-world scalability, complete privacy protection or clinical deployment readiness.

### 6.1 Strengths of the proposed system

- Enables efficient use of labeled and unlabeled ECG data through semi-supervised learning.
- Reduces the need for manually annotating data.
- Reduces the need for direct sharing of raw ECG data across clients through federated learning.
- Improves feature representation by using adaptive feature fusion of multi-level features.
- Uses adaptive confidence-based pseudo-labeling for enhancing learning reliability.
- Compares the performance of various machine learning models.
- Uses weighted federated aggregation for better updates from model in federated learning.

### 6.2 Pseudo code for the proposed system

The overall workflow of the proposed semi-supervised federated learning framework is summarized in Algorithm 1.

ALGORITHM 1 **Semi-supervised federated learning framework for ECG-based arrhythmia detection.**

Input: MIT-BIH ECG dataset D
Output: Global Model G and Evaluation Metrics
BEGIN
1. DATA LOADING
    Load ECG records from dataset D
    Extract annotations (r-peak positions and labels)
2. HEARTBEAT SEGMENTATION
    FOR each record r in D DO
    FOR each valid heartbeat index i DO
    Extract segment of fixed window size (200 samples)
    Store segment and corresponding label
    END FOR
    END FOR
3. LABEL PROCESSING
    Convert labels into binary classes:
    Normal –> 0
    Arrhythmia –> 1
4. CLASS BALANCING
    Apply SMOTE to balance class distribution
5. FEATURE EXTRACTION
    FOR each ECG segment s DO
    Extract raw signal features (200 values)
    Compute statistical features: Mean, standard deviation, max, min, energy, skewness, kurtosis
    Extract wavelet features using discrete wavelet transform
    END FOR
6. FEATURE NORMALIZATION
  Standardize raw, statistical and wavelet features separately
7. ADAPTIVE FEATURE FUSION
    Compute group-level variance after standardization:
    var_raw = variance(raw_features)
    var_stat = variance(statistical_features)
    var_wave = variance(wavelet_features)
    Compute total variance:
    total_variance = var_raw + var_stat + var_wave
    Compute global fusion weights:
    w_raw = var_raw/total_variance
    w_stat = var_stat/total_variance
    w_wave = var_wave/total_variance
    Generate fused feature vector:
    F = [w_raw * raw_features, w_stat * stat_features, w_wave * wavelet_features]
8. DATA SPLITTING
    Split dataset into:
    Training set (80%) and Test set (20%)
    Split training set into:
    Labelled set (20%) and Unlabelled set (80%)
9. FEDERATED LEARNING SETUP
    Divide training data across N clients
    FOR each client i DO
    Split client data into:
    Labelled data D_l^i
    Unlabelled data D_u^i
    END FOR
10. INITIALIZE GLOBAL MODEL G
11. FEDERATED TRAINING WITH SEMI-SUPERVISION
    FOR each communication round r=1 to R DO
         *FOR each client i=1 to N DO*
     	*//Local Training*
     	*Train Random Forest model M_i on D_l^i*
     	*//Adaptive Pseudo Labelling*
     	*Predict probabilities for D_u^i*
     	*Compute confidence scores*
     	*Compute adaptive threshold:*
     		τ = mean(confidence) + 0.5 * std(confidence)
     	*FOR each sample x in D_u^i DO*
     		*IF confidence(x) >= τ THEN*
     			*Assign pseudo label y*
     			*Add (x,y) to D_l^i*
     		*END IF*
     	*END FOR*
     	*//Retraining*
     	*Retrain model M_i on updated labelled dataset*
     	*Send model predictions to server*
         *END FOR*
        *//Weighted Federated Aggregation*
         *FOR each client i DO*
     	*weight_i = size(D_l^i)/total_data*
         *END FOR*         *         Aggregate predictions:*
     	G = Σ (weight_i * M_i predictions)
    END FOR
12. MODEL EVALUATION
    Compute evaluation metrics: Accuracy, Precision, Recall, F1-score, ROC curve, AUC score
13. OUTPUT RESULTS
    Compare performance across: Centralized model, Federated (IID) and Federated (Non-IID)
END

**Figure 2:**
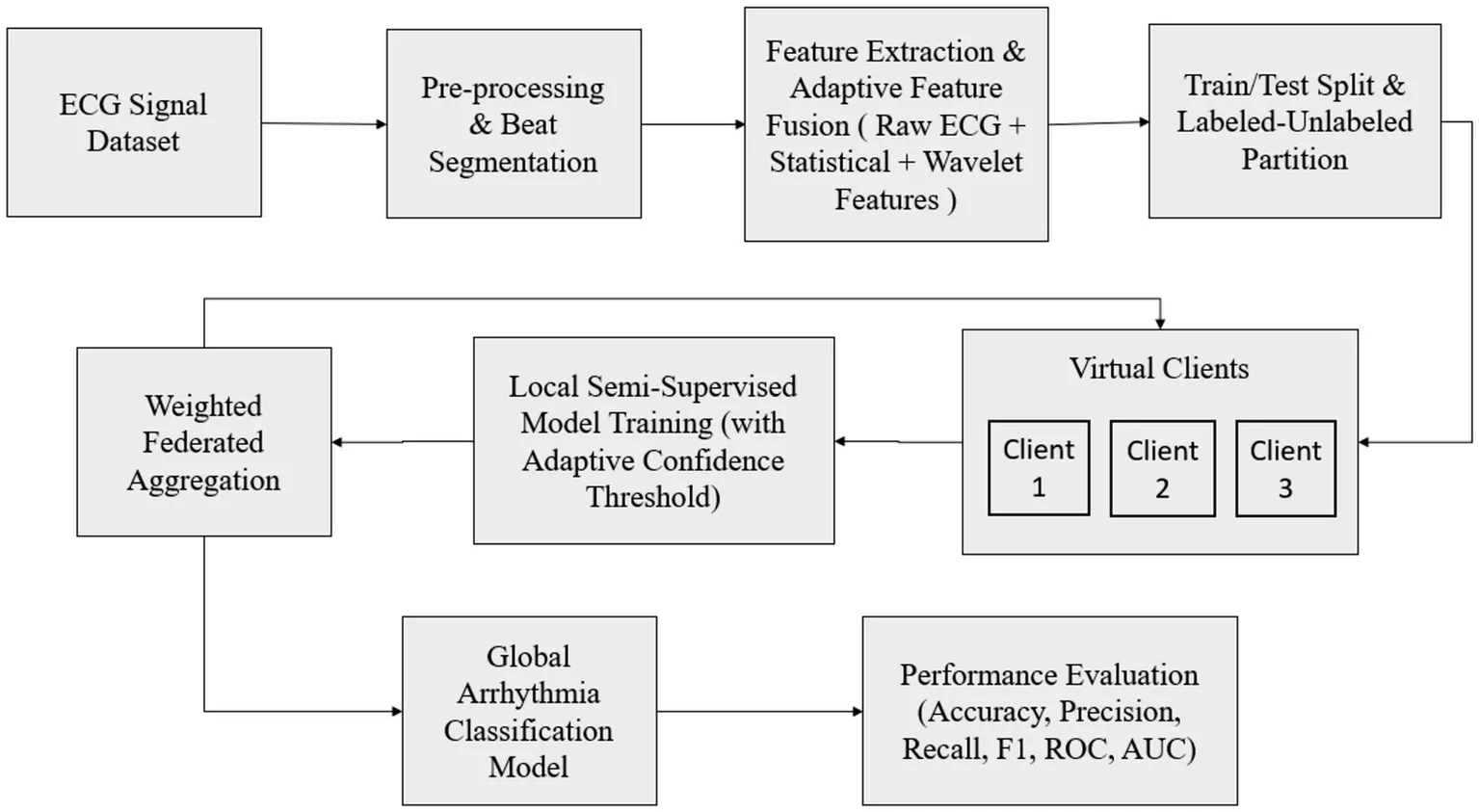
System architecture for proposed system.

The system architecture shown in Figure 2, shows the overall workflow of how the proposed system framework works. It is a structured pipeline in which there is an integration of data pre-processing, feature extraction, semi-supervised learning and federated learning. As the first step, the ECG signal dataset is made to undergo pre-processing and beat segmentation. This helps in removing the noise and also isolating the heartbeat signals individually. Using these signals that are segmented, extraction of different features was done. The features extracted include the raw ECG features, statistical features and wavelet-based features. An adaptive feature fusion is applied to these extracted features to combine them. This helps in creating a comprehensive and informative representation of features. The dataset that is processed is then divided into two, one of the training set and the other the testing set. The training set is then divided into labeled and unlabeled sets so that it can be used for semi-supervised learning. After this, a federated learning environment set up is simulated by performing distribution of data across multiple number of virtual clients. Then each of the client will undergo local model training by using a semi-supervised approach along with an adaptive confidence threshold that is used for pseudo labeling. These models that are trained locally are then aggregated by using a strategy called weighted federated aggregation that in turn forms the global arrhythmia classification model. Then standard metrics like accuracy, precision, recall, f1-score, ROC and AUC are used to calculate the performance of this global model. This kind of architecture framework ensures that there is efficient utilization of data, privacy preservation, improvement in classification performance by performing integration of multiple advanced learning techniques.

## 7 Implementation

This chapter discusses the practical implementation of the system that was created for arrythmia detection which is formed by the integration of semi-supervised learning and federated learning. The system has many components including ECG signal processing, extraction of features, adaptive feature fusion, semi-supervised learning and federated learning. Each of the modules are clearly explained with the help of code snippets. To start with, all the implementation is done in Google Colab using Python programming language. Google Colab’s dynamic runtime is based on Ubuntu Linux and provides access to NVIDIA Tesla T4 with 16GB VRAM. So, GPU T4 was opted for hardware accelerator. It provides high disk environment and faster training as well. Also, it includes use of many libraries including pandas, matplotlib, numpy, wfdb, PyWavelets, scikit-learn etc. Pandas and Numpy are used for handling data and preprocessing. WFDB is used in the loading of ECG signals and its annotations from the dataset. PyWavelet is used to do extracting of wavelet-based features from the ECG signals. Scikit-learn helps to implement various the machine learning models. SMOTE is used to handle the imbalance in the class. Matplotlib is used for doing the visualization tasks like distribution plots, graphs, matrices etc. The complete implementation has different stages of process. All the important codes that are involved in each module of the project are shown as follows.

### 7.1 Dataset collection and segmentation

**Figure 3:**
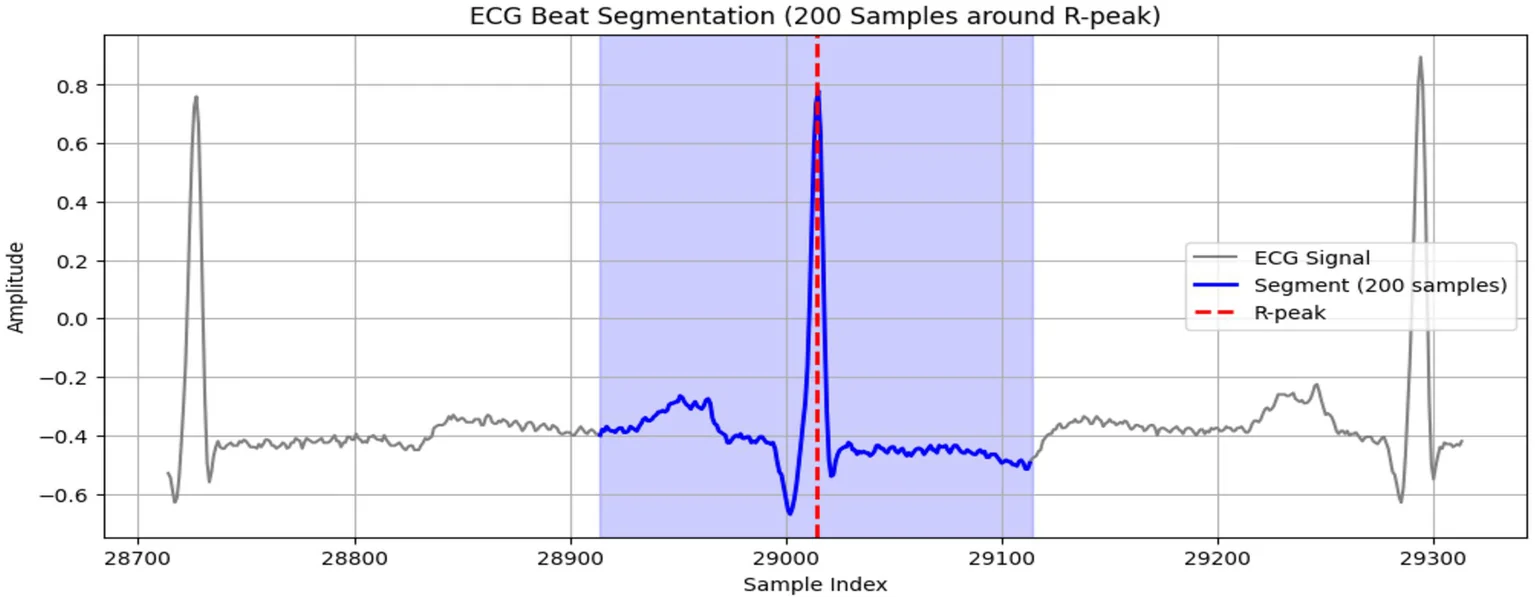
ECG signal segmentation.

**Figure 4:**
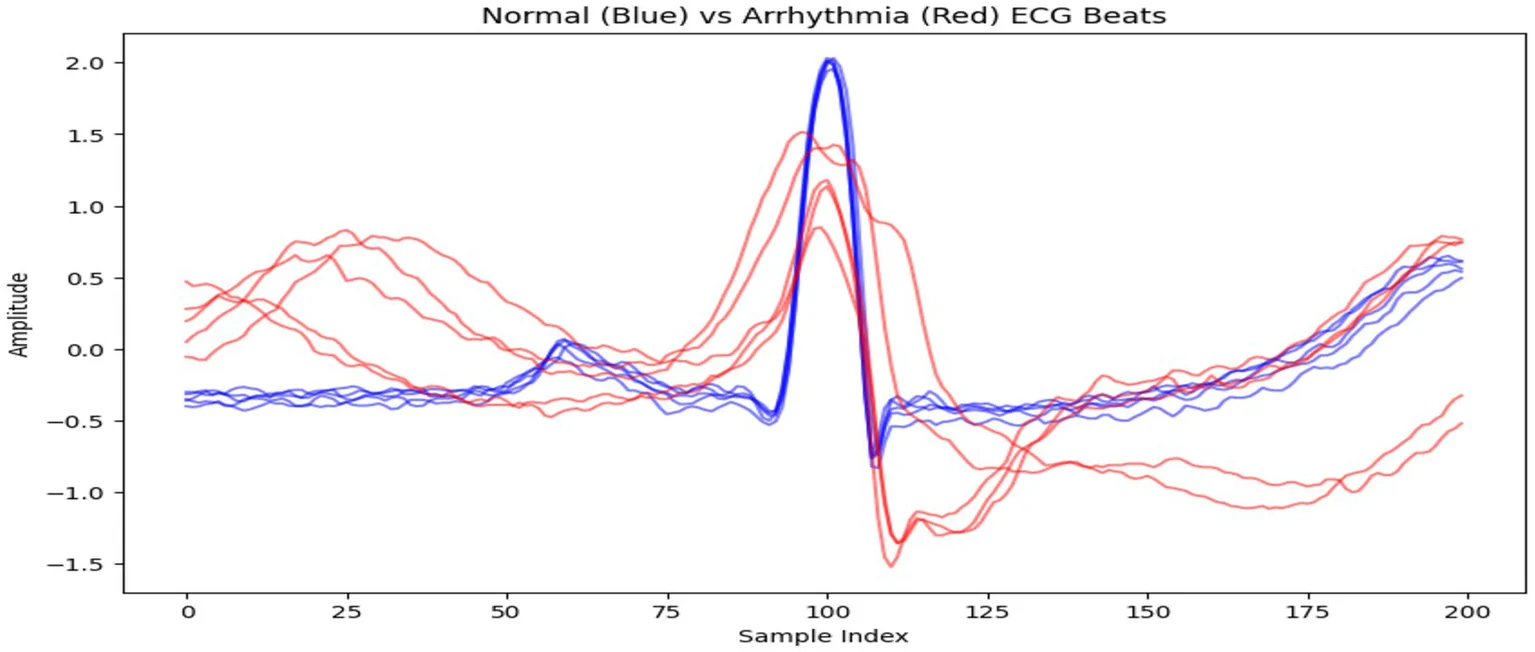
Normal vs. arrhythmic ECG beats.

The dataset chosen for the study was one of the most used and the benchmark dataset that is widely recognized called MIT-BIH Arrhythmia Dataset. This dataset consists of 48 ECG recordings stored in .dat format each sampled at 360 Hz and contains approximately 650,000 signals per record as shown in Table 2. The corresponding annotation files stored in .atr files are used to determine the type and position of each of the heartbeat. Annotation files are significant in a way that it helps in identification and extraction of meaningful cardiac patterns from the continuous ECG signals. The ECG signals are accessed and properly processed with the help of a library called WFDB. This library has efficient tools that has the ability to read waveform data and its associated annotations or labels. These annotation symbols are used to find out the locations of valid heartbeats like [‘N’, ‘L’, ‘R’, ‘A’, ‘V’, ‘F’, ‘J’, ‘E’, ‘a’, ‘j’, ‘f’]. During ECG beat extraction, only those heartbeat annotations that are clinically relevant were considered for further processing. Those annotation symbols that corresponded to the non-beat events, rhythm change makers, pacing artifacts, signal quality indicators, unknown beats or other non-classifiable events were excluded from analysis. This step was carefully carried out to ensure that the dataset consists of only the valid heartbeat segments that are suitable for binary arrhythmia classification. So WFDB helps in reading these annotation files and also filter out those annotations that are found to be irrelevant or noisy. This process ensures data quality. After identifying the valid positions of the heartbeats, individual heartbeat samples are segmented from these continuous ECG signals as shown in Figure 3. A fixed size window of 200 samples is used to extract each heartbeat by centering around the R-peak and this window size would help capture the surrounding region of the ECG signal which would include the P wave, QRS complex, and the T wave. This ensures that a consistent structure is maintained by all the samples as it is very essential for extracting features and training models that follows. The output of this module is a set of clean and uniformly segmented signals of heartbeat as shown in Figure 4. This forms the base for the other stages in the framework that follows.

**Table 2:** Dataset description.

Parameter	Value
Dataset	MIT-BIH
Records	48
Sampling frequency	360 Hz
Approximate signal strength	650,000 samples
Total beats extracted	102,385
Segment size	200

### 7.2 Data preprocessing and feature engineering

**Figure 5:**
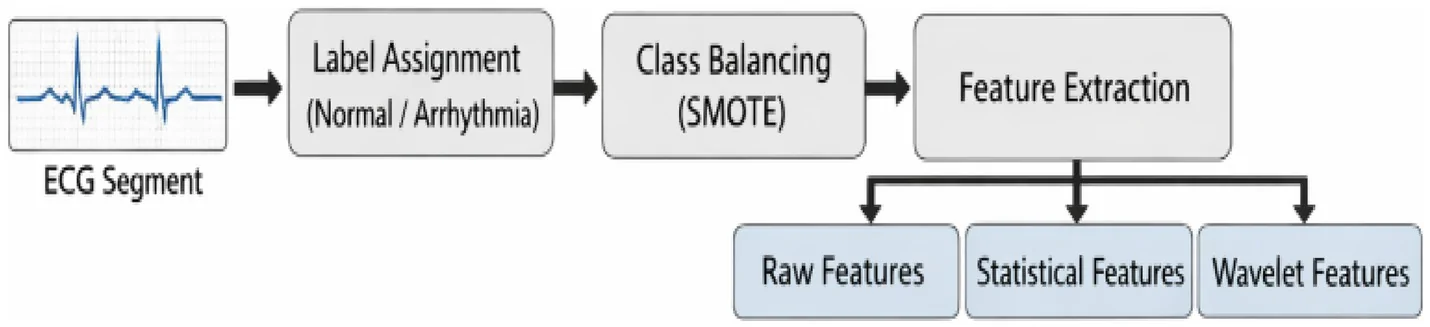
Preprocessing and feature engineering pipeline for ECG signal preparation.

Data preprocessing and feature engineering is performed to make sure that the segmented heartbeats are prepared for an effective training of the model. First, the heartbeats that are extracted are properly organized and then the appropriate class labels are assigned to these segments depending upon their annotation type. A binary classification is carried out in which the normal heartbeats are labeled as class 0 and differentiated from the arrhythmic ones which are grouped as class 1. This simplification ensures that there is improved model learning and interpretation. The ECG dataset chosen has high class imbalance with a higher number of normal samples (75,028) when compared to arrhythmic samples (27,357). The imbalance between the two classes is corrected using SMOTE to ensure that there is no bias by the model toward the majority class as shown in Figure 5. After SMOTE, a balanced dataset of 150,056 samples is formed. This helps in creating an unbiased and reliable learning process. After preprocessing, feature engineering is also performed that helps in creating meaningful representations by transforming ECG signals. Various types of features are extracted in order to identify the different characteristics of the signal. The features extracted include 200 samples of raw signal features that is used to show the original waveform, seven statistical features (mean, standard deviation, maximum, minimum, signal energy, skewness and kurtosis) that helps in summarizing the properties of the beat signal and lastly eight wavelet-based features that helps in identifying the frequency-domain information that is extracted using Discrete Wavelet Transform (DWT) using the Daubechies (db4) wavelet at level 3 decomposition. These features are combined to form a system that would ensure a comprehensive representation of ECG signals that can help in improving the effectiveness of the classification in the following stages (Tables 3, 4).

**Table 3:** Dataset size before and after SMOTE.

Class	Before applying SMOTE	After applying SMOTE
Normal (0)	75,028	75,028
Arrhythmia (1)	27,357	75,028
Total	102,385	150,056

**Table 4:** Details of features extracted.

Feature type	Number of features
Raw signal	200
Statistical	7
Wavelet	8
Total	215

### 7.3 Feature fusion mechanism

**Figure 6:**
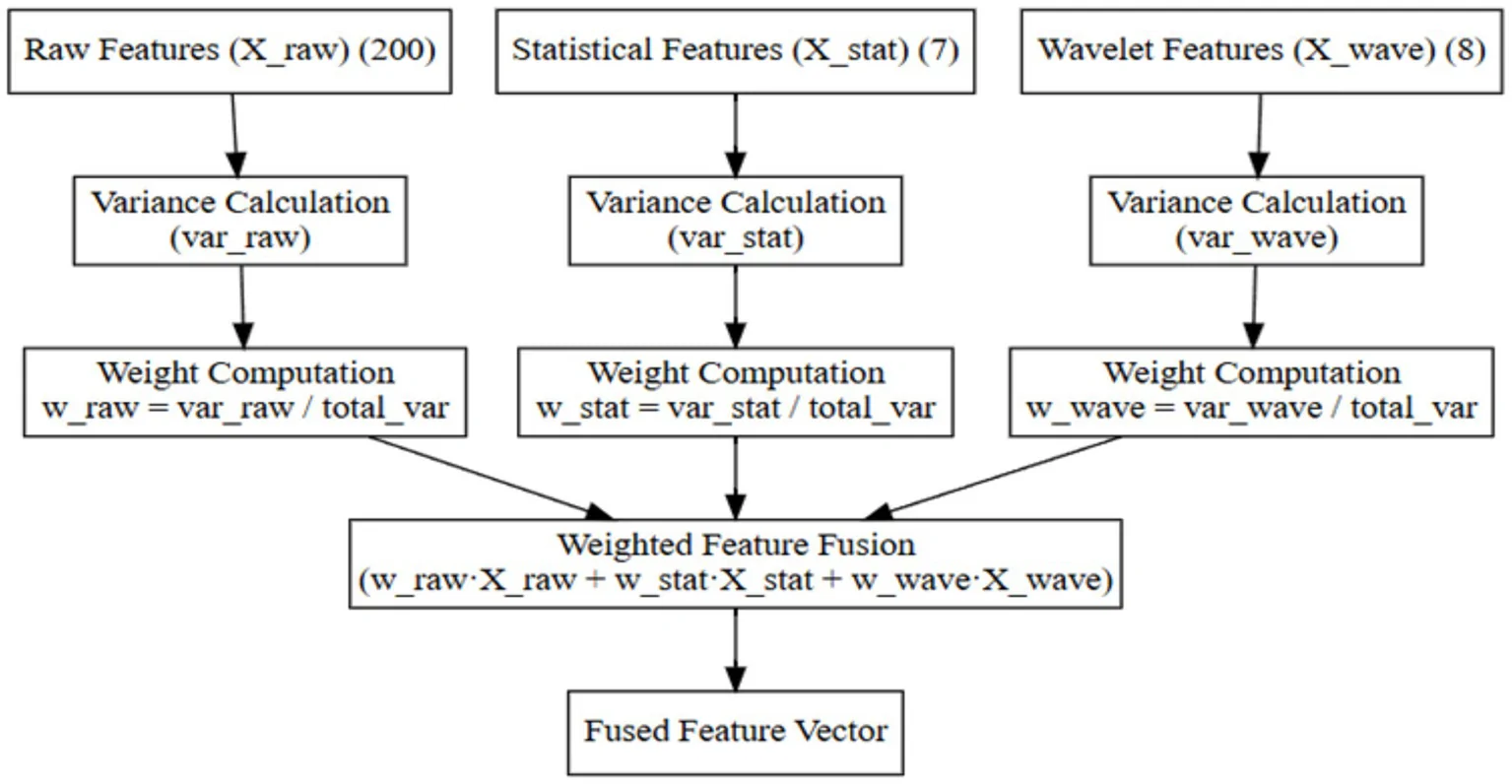
Adaptive feature fusion using variance-based weighting after feature standardization.

The aim of this module is to create a unified and more informative representation system by combining the different features that were extracted from the ECG signals. It is a significant step to integrate the features, i.e., raw, statistical and wavelet-based features effectively instead of treating them independently. This proper combining has to be done as each of the feature types has the ability to identify different features of the ECG signal. This process helps in improving the classification performance. Normalization is applied before combining the features as it is necessary to make sure that all the feature groups are on a comparable scale. StandardScaler is applied independently to each feature group, transforming them to have zero mean and unit variance that prevents any single feature type from dominating the learning process. Once the normalization is completely done, a mechanism called adaptive feature fusion mechanism is used to integrate the features in a meaningful manner. Importance is assigned to each of the feature group by using a variance-based weighting approach instead of directly combing all the features as shown in Figure 6. After standardization, the variance of each feature group is calculated to get one group-level variance value for the three feature groups. The weights for each of these feature groups are calculated using the following formula: w_i = variance_i/total variance, where total variance is the sum of the three group-level variance values. These weights are calculated globally during preprocessing, even before the training data are distributed among the federated clients and remain fixed during the federated training rounds. Here variance is used as a weighting criteria and is not at all considered as a direct measure of the discriminative information contained in a feature group. High variance may also arise from noise, artifacts, outliers or inter-patient variability. No separate analysis was performed in the present implementation to establish that a highly weighted feature group provides greater class-discriminatory information than a lower-weighted feature group. This type of adaptive fusion technique helps the overall feature quality to be enhanced and also helps the model in capturing complex patterns in the ECG. So, a fused feature vector will be the output of this module while maintaining the dimensionality of 215 features. It retains the contributions of the individual feature types in the fused representation for subsequent classification.

When compared to the conventional and existing feature fusion approaches that directly perform concatenation of multiple feature sets or assigning fixed weights, the proposed system uses an adaptive feature fusion technique where it computes variance-based adaptive weights for raw ECG signals, statistical features and wavelet features before fusion. This strategy of data-driven weighting assigns different relative contributions to the raw ECG, statistical and wavelet feature groups at the same time preserving complementary information from multiple feature domains. This results in a richer representation for subsequent classification within the semi-supervised federated learning framework.

### 7.4 Semi-supervised learning

This module aims to enhance the system’s ability to learn by making use of both labeled and unlabeled ECG data. It is expensive and time consuming to get labeled data especially in real-world scenarios but at the same time there is a large amount of data that is left unlabeled and is readily available. So, to overcome this challenge, a semi-supervised learning paradigm is used that allows the system to learn in an effective manner even when the number of labeled data is limited. The dataset is divided into training (80%) and testing (20%) sets using stratified sampling. Then the training dataset consisting of 120,044 samples is then divided into labeled (20%) and unlabeled (80%) samples to simulate a semi-supervised learning environment. This configuration was done to reflect a realistic scenario where only a limited amount of the data that is available is labeled whereas the remaining data is kept as unlabeled for pseudo label generation. This results in 24,008 labeled samples and 96,036 unlabeled samples as shown in Table 5. The model is made to learn the basic patterns of the ECG signals using the labeled data. Once the model is done training, it is made to predict the labels or annotations for the unlabeled data. This process of labeling is termed as pseudo labeling. In this kind of approach, the model will be predicting the labels for the data that was not previously seen.

**Table 5:** Dataset split.

	Category	Samples
Initial split	Total dataset	150,056
	Training	120,044
	Testing	30,012
Semi-supervised split (from training data)	Labeled	24,008
	Unlabeled	96,036

**Figure 7:**
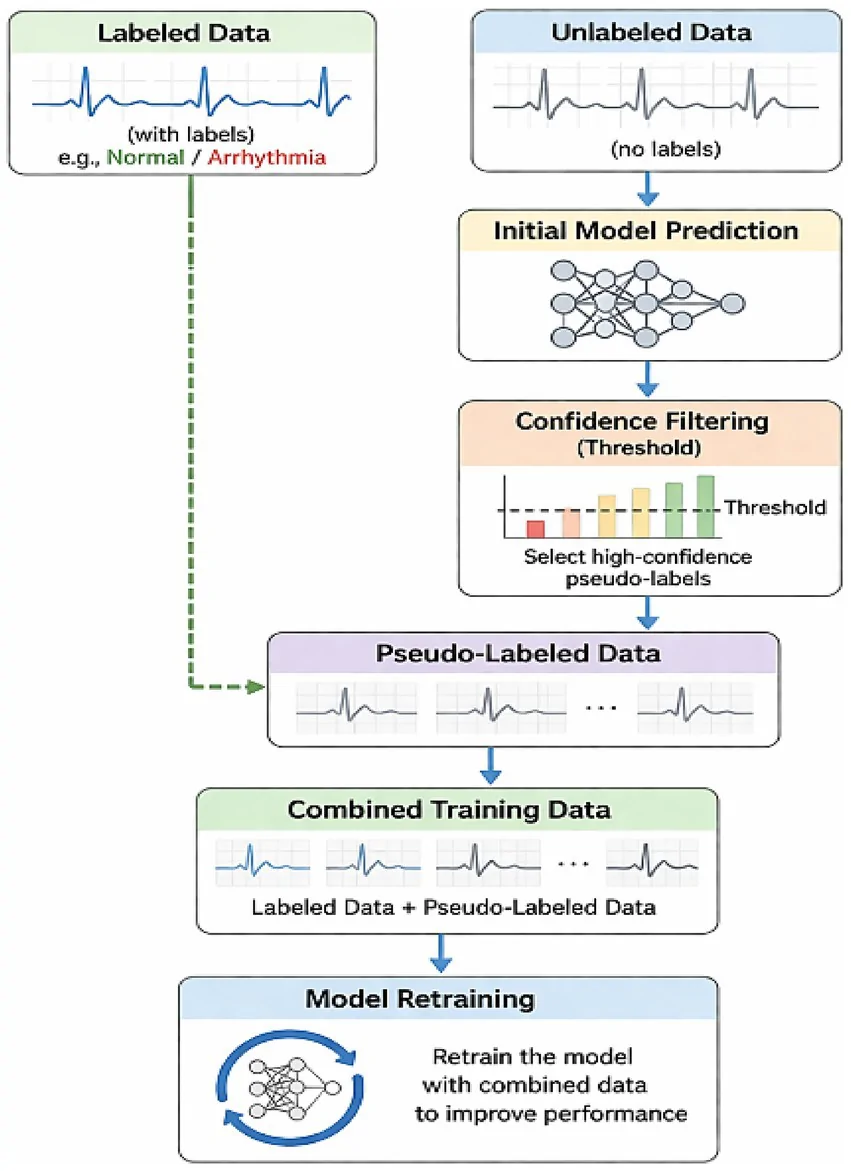
Semi-supervised learning using pseudo labeling with adaptive confidence threshold.

To make sure that reliability is maintained, the pseudo labeled samples are selected using an adaptive confidence-based threshold. The formula used to calculate threshold is as threshold = mean (confidence) + 0.5*std. (confidence). The value of the threshold is capped at a maximum value of 0.99. Only those predictions that have higher confidence value will be added to the training dataset. Using this technique of dynamic threshold, uncertain predictions can be removed out and the quality of the augmented dataset can be improvised. Once the pseudo labels are selected, it is then combined along with the labeled data and forms an expanded dataset as shown in Figure 7. The expanded dataset is used to train the model again, so that the model can now learn from a larger set of data and potentially improve its performance on unseen test samples. This module helps in improving the performance mainly in scenarios where the data is limited.

**Figure 8:**
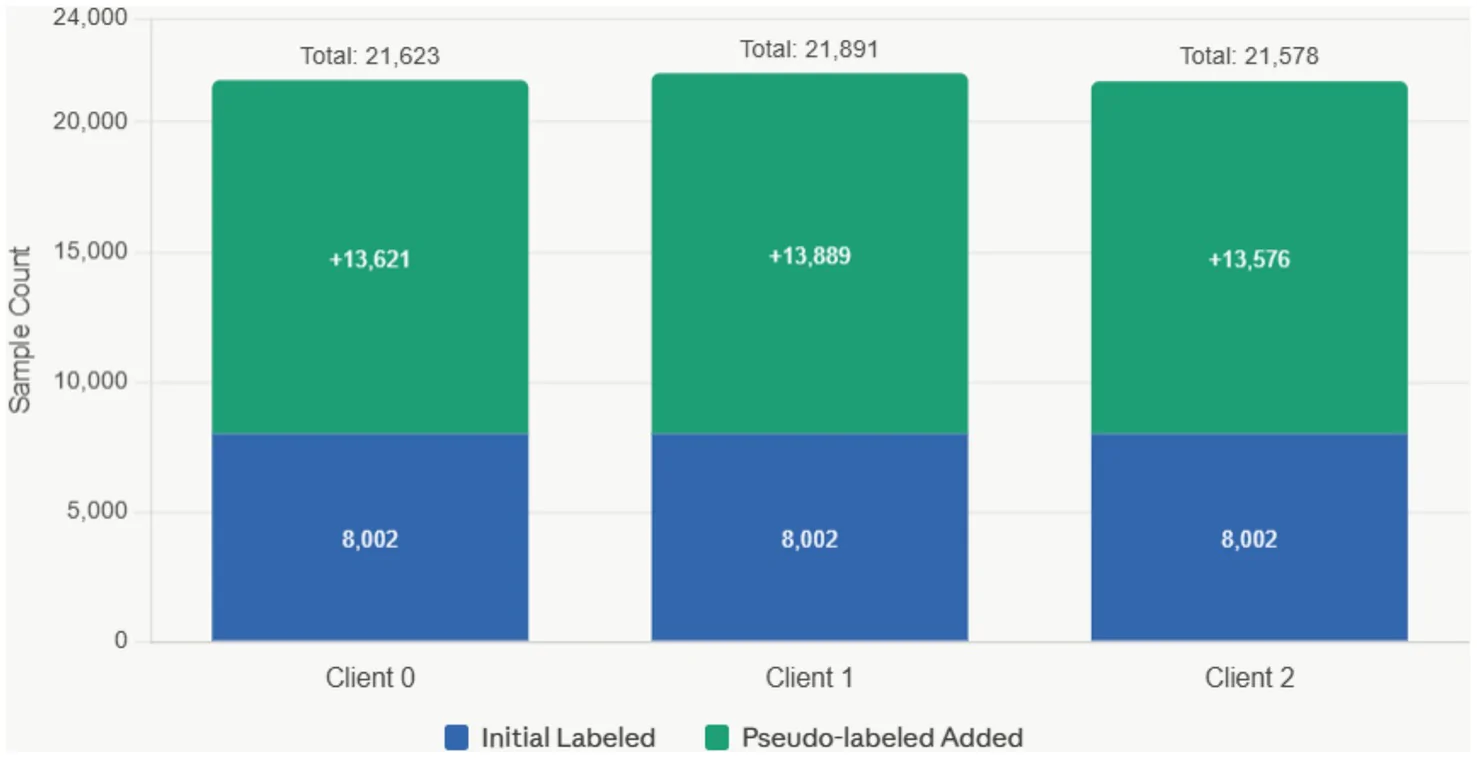
Pseudo label expansion.

As seen in Table 6 and Figure 8, after pseudo-labeling is done, there is a significant increase in the annotated data. Only samples with high threshold are selected by use of adaptive threshold. This helps in improving learning without the need for manual labeling.

**Table 6:** Pseudo label expansion.

Client	Initial labeled	Pseudo-labeled added	Final labeled
Client 0	8,002	13,621	21,623
Client 1	8,002	13,889	21,891
Client 2	8,002	13,576	21,578

### 7.5 Federated learning framework

This module aims to build a distributed model training method by making sure that privacy is preserved. In the traditional learning approaches, the storage of all data is in a centralized location. In applications like that of healthcare, it raises serious concerns like privacy and security concerns when it comes to sharing of sensitive patient data across systems. Federated learning plays its role here. The training dataset is split across various multiple clients. Each client will be simulating an independent data source. So, each client will be training using its own data instead of sharing the raw ECG data. This helps in keeping the raw ECG data within the respective simulated client instead of directly sharing the raw signals with other clients or the aggregation process. This might help in reducing the data exposure risks that are associated with centralized raw-data sharing. Federated learning alone will not provide a guarantee of complete privacy as the model parameters or updates that are exchanged during federated training may still present privacy risks.

The proposed system tries this mechanism in both homogeneous and heterogenous data distribution scenarios. There are cases where all clients will be having the same type of data distribution as in Table 7 and also other real-world settings where the data will be significantly varying across various sources as in Table 8. The system will become more robust and adaptable to practical healthcare environments when it is made to handle both the type of distributions. Once the local training is done in each of the clients, the models received from each of them is used for aggregation. This approach of distributed learning allows for privacy as well as collaborative learning across various data sources without the need for sharing of the data directly.

**Table 7:** Number of labeled and unlabeled data.

Client	Total	Labeled	Unlabeled
Client 0	40,014	8,002	32,012
Client 1	40,014	8,002	32,012
Client 2	40,014	8,002	32,012

**Table 8:** Distribution of data among clients (non-IID).

Client	Normal	Arrhythmia
Client 0	16,005	4,001
Client 1	4,001	16,005
Client 2	40,016	40,016

**Figure 9:**
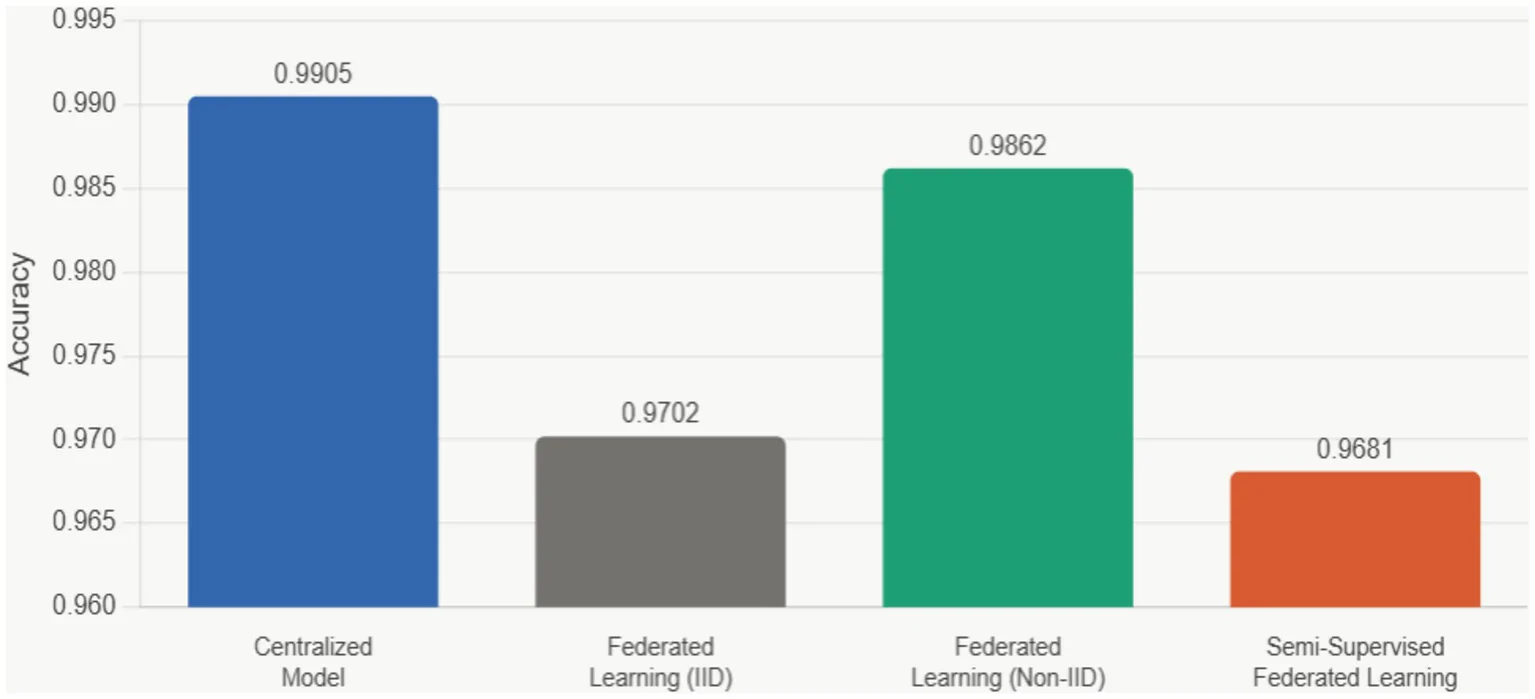
Accuracy comparison across centralized and federated models.

As seen in Table 9 and Figure 9 the highest accuracy is shown by centralized model as it has the access to complete data. The accuracy slightly reduces when it comes to simulated federated learning while the raw ECG data remains locally held by the simulated clients rather than being directly shared. Federated learning in a non-IID setup helps in achieving an accuracy that is closer to that of the centralized model. This shows that the model can maintain its performance under the simulated heterogenous data distribution. Semi-supervised learning also makes use of unlabeled data with minimal performance drop.

**Table 9:** Overall model performance (random forest).

Model type	Accuracy
Centralized model	0.9905
Federated learning (IID)	0.9702
Federated learning (Non-IID)	0.9862
Semi-supervised federated learning	0.9681

**Figure 10:**
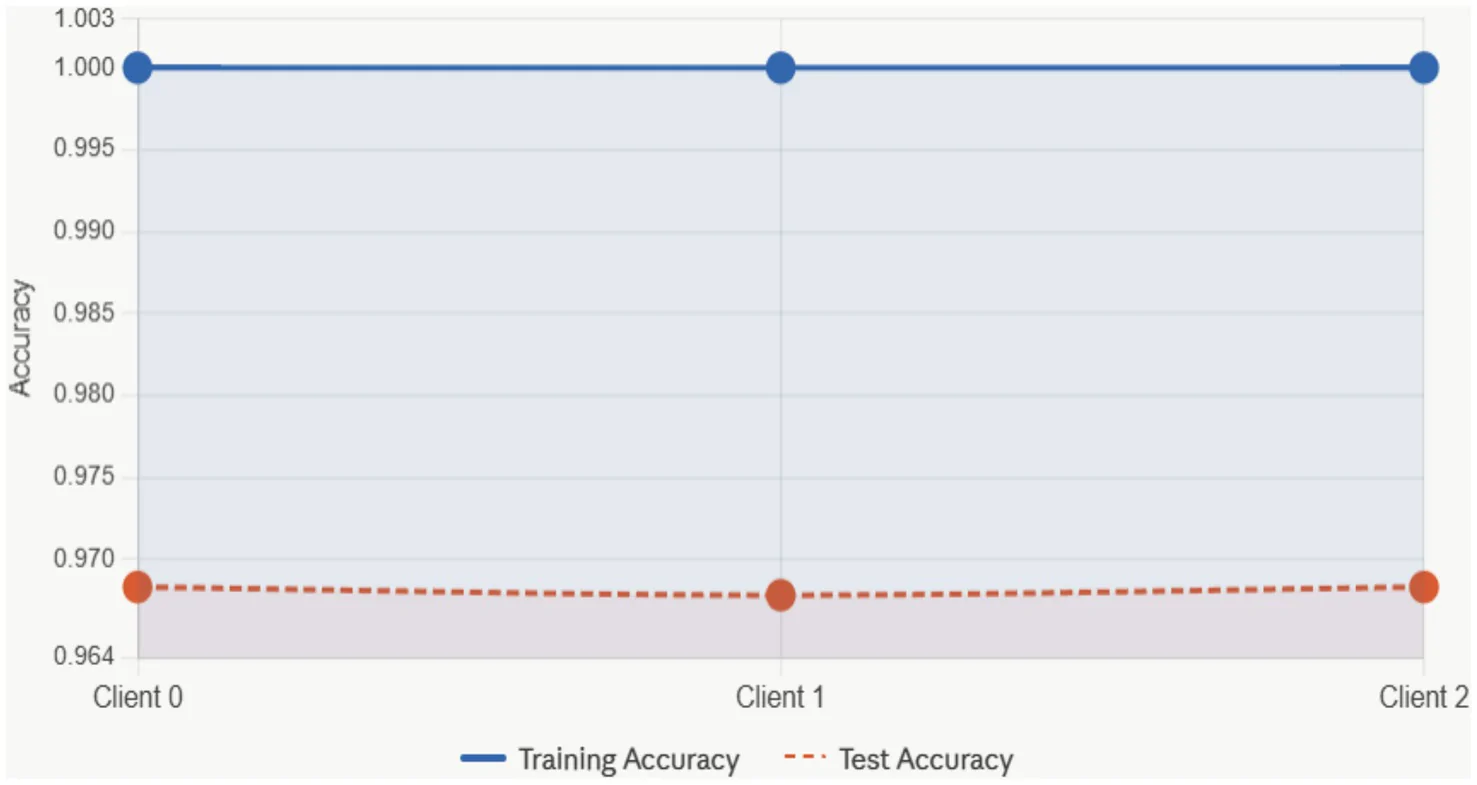
Line chart showing the client model performance.

As seen in Table 10 and Figure 10, training accuracy is perfectly achieved by all the client models. This shows strong learning capability. The test accuracy is maintained consistently across the clients (~96.8%), which indicates that there is balanced data distribution. Clients show similar performance which ensures there is stable federated aggregation.

**Table 10:** Client model performance.

Client	Training accuracy	Test accuracy
Client 0	1.000	0.9683
Client 1	1.000	0.9678
Client 2	1.000	0.9683

### 7.6 Model training and aggregation

**Figure 11:**
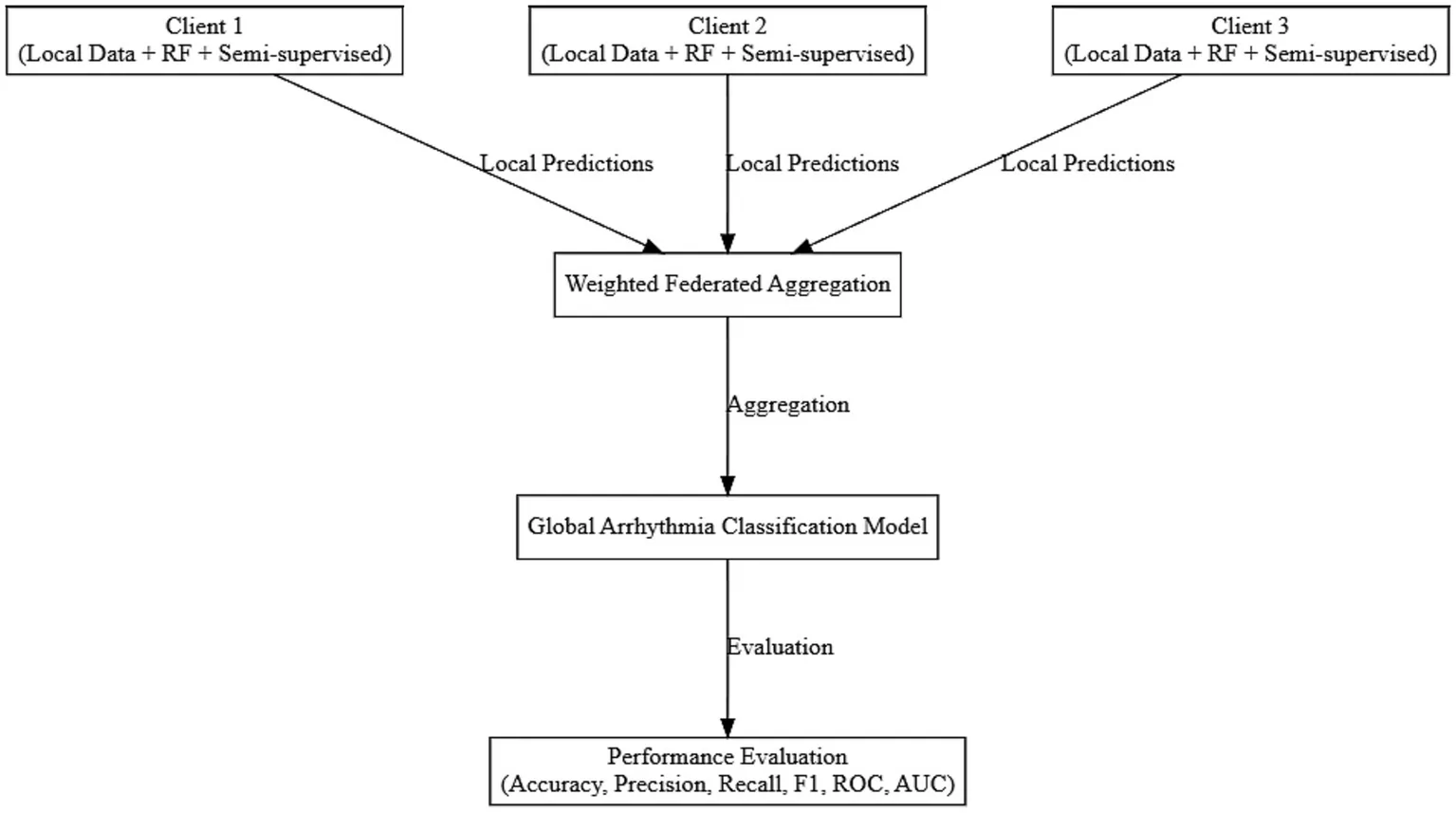
Federated model training and aggregation process for global arrhythmia classification.

In this module, the machine learning models are trained at the client side and the outputs from each client is combined to form the unified global model. Each of the client will be using its own data to train. This data will include both labeled and pseudo-labeled data that was obtained by doing semi-supervised learning. This will help in allowing the model to train and learn using both reliable data that is labeled and the high confidence predictions. Machine learning models including Random Forest, SVM, KNN, AdaBoost, Gradient Boosting and MLP are implemented within the system. From these, the Random Forest model is chosen as the primary model in the proposed framework because it shows robustness and has the ability to handle complex feature representations. Random Forest can model complex non-linear relationships and it exhibits resistance to overfitting through ensemble learning. Since there are heterogenous statistical, temporal and wavelet-based characteristics in the extracted ECG feature set, this makes Random Forest suitable for handling high dimensional and mixed type features without the need for extensive feature scaling. Also, the model’s computational efficiency and interpretability make the model an apt choice for federated learning environments in which reliable performance across distributed clients is essential. The model is configured with 100 estimators, a fixed random state (42) and parallel processing. In addition to this, to provide a comprehensive evaluation, other ML models including SVM, KNN(k = 5), AdaBoost (100 estimators), Gradient Boosting and Multi-Layer Perceptron are trained to do comparative analysis and evaluate performance. Once local training is done, prediction outputs are generated by each of the client using its trained model. These outputs produced undergoes a weighted aggregation strategy and combines as shown in Figure 11. The contribution of each client is determined based on the number of labeled samples. The formula used to calculate the weight given to each client is w_i = size_i/total size where size_i represents the number of labeled samples in client i. Here, each of the client does its own contribution to the final prediction depending upon its relative importance. This makes sure that clients that are more reliable or has more data will show a greater influence on the global model. A global arrhythmia classification model is formed from the aggregated output by integrating the knowledge that is taken from all the clients. The global model prediction is computed as Weighted Probability = ∑ (w_i x P_i) where P_i is the prediction probability from client i. So, for the final prediction, the class that will show the highest probability will be selected. This kind of collaborative aggregation helps to combine the information learned by the participating clients and also supports the evaluation under different simulated data distributions. The aggregation process is then repeated again for a multiple number of communication rounds, here it is taken as 5 rounds. The models are evaluated under multiple configurations including centralized learning, federated learning with IID distribution, federated learning with non-IID distribution and semi-supervised federated learning. This comprehensive evaluation facilitates comparison of model performance and helps identify the most suitable approach for arrhythmia detection within the proposed framework. The final model obtained is then used for doing the remaining steps like evaluation and analysis.

**Figure 12:**
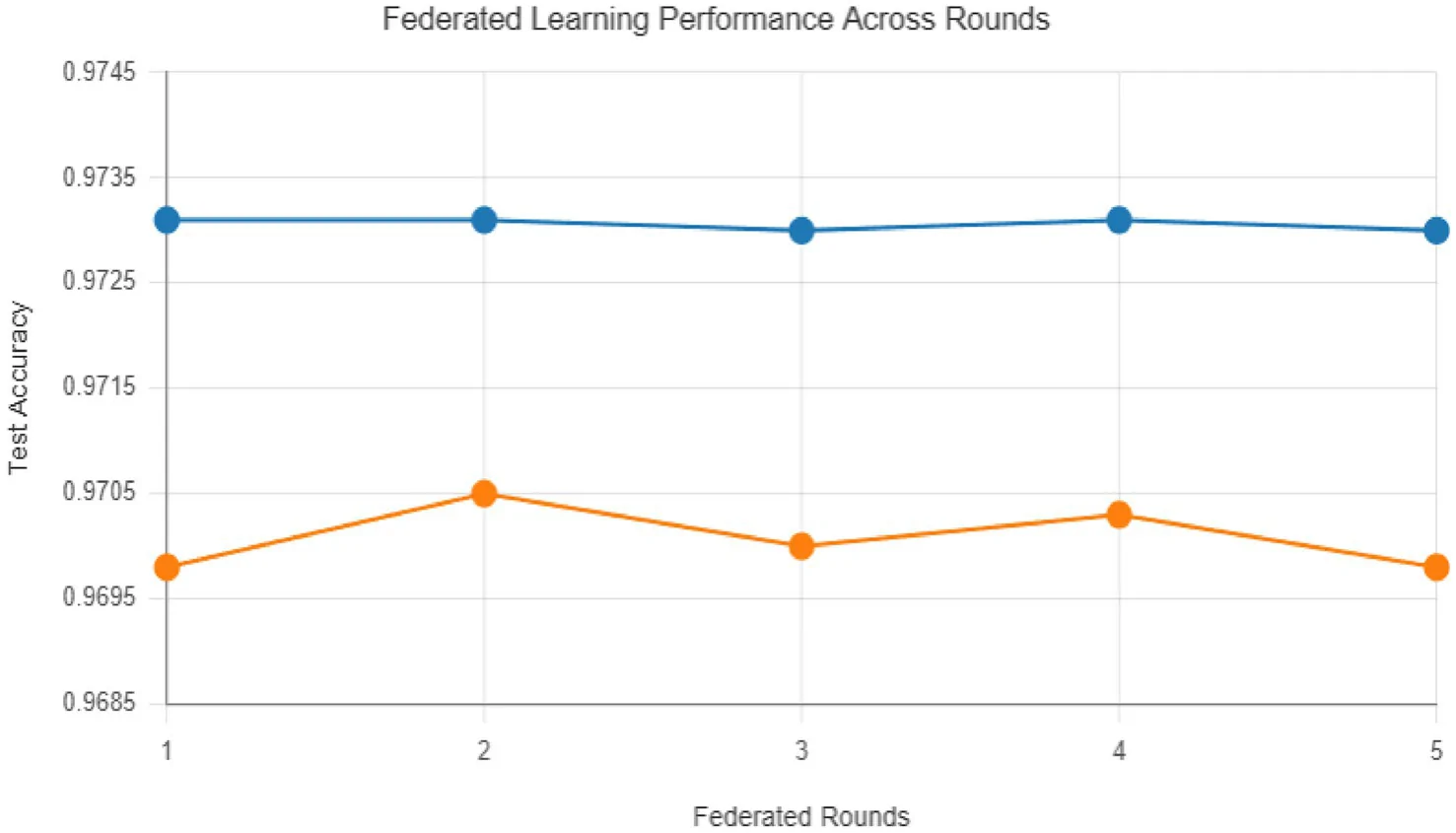
Federated learning performance across rounds.

The performance of the model is stable across all the rounds as shown in Table 11 and Figure 12. There are slight fluctuations that shows that there is consistent convergence. This shows that there is reliability in the federated training process.

**Table 11:** Federated learning across rounds.

Round	Training accuracy	Test accuracy
Round 1	0.9731	0.9698
Round 2	0.9731	0.9705
Round 3	0.9730	0.9700
Round 4	0.9731	0.9703
Round 5	0.9730	0.9698

### 7.7 Evaluation and visualization

This module involves the evaluation of the performance of the proposed arrhythmia detection system by making use of the final global model. The model is tested by giving unseen ECG data and seeing its ability to correctly classify as either normal or arrhythmic heartbeats. The standard metrics like accuracy, precision, f1-score, recall, ROC, AUC are used for measuring the performance. Also, other visualization techniques like confusion matrix and ROC curve are also used so that the model’s behavior and performance can be easily understood. This module is used to validate the proposed system’s effectiveness and reliability for detection of arrhythmia.

Table 12 shown shows a comparison of various machine learning models used for evaluating the performance of the proposed federated learning framework. Ensemble and neural network-based models show better performance and depicts the importance and effectiveness in handling ECG signal classification.

**Table 12:** Performance comparison of different models under federated learning framework.

Model	Accuracy	Precision	Recall	F1-score
Random forest	0.970012	0.970653	0.970012	0.970002
SVM	0.952519	0.953310	0.952519	0.952498
KNN	0.969845	0.970259	0.969845	0.969839
AdaBoost	0.897208	0.897289	0.897208	0.897203
Gradient boosting	0.948154	0.949065	0.948154	0.948128
MLP	0.980574	0.980662	0.980574	0.980574

**Figure 13:**
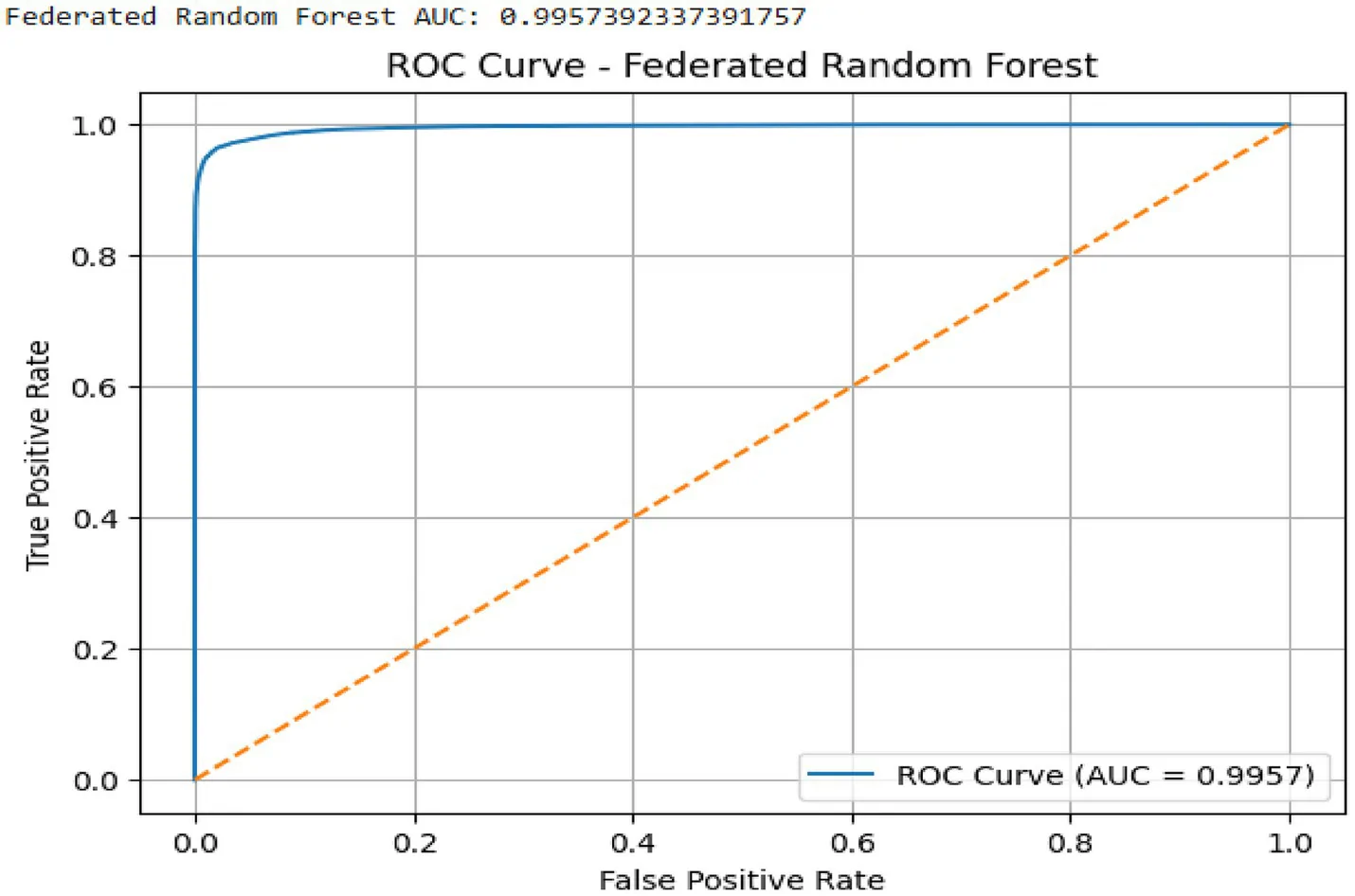
ROC curve for Federated Random Forest Model.

**Figure 14:**
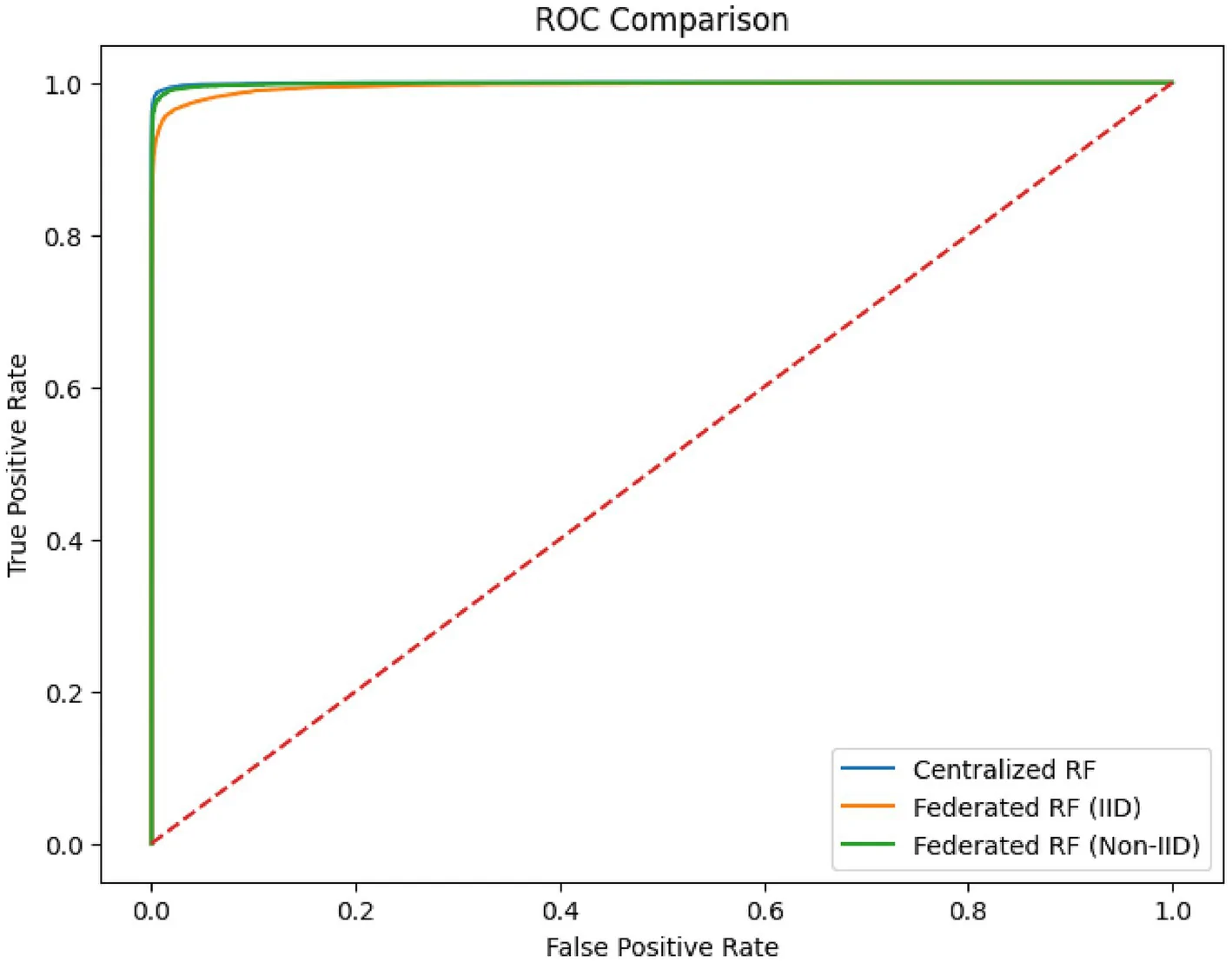
ROC curve between Centralized and Federated models.

As seen in Figures 13 and 14, there is a good classification capability as the model shows high AUC value. Federated model achieves a performance that is comparable with that of centralized model. There is a strong separation between the classes as shown by the ROC curves.

**Figure 15:**
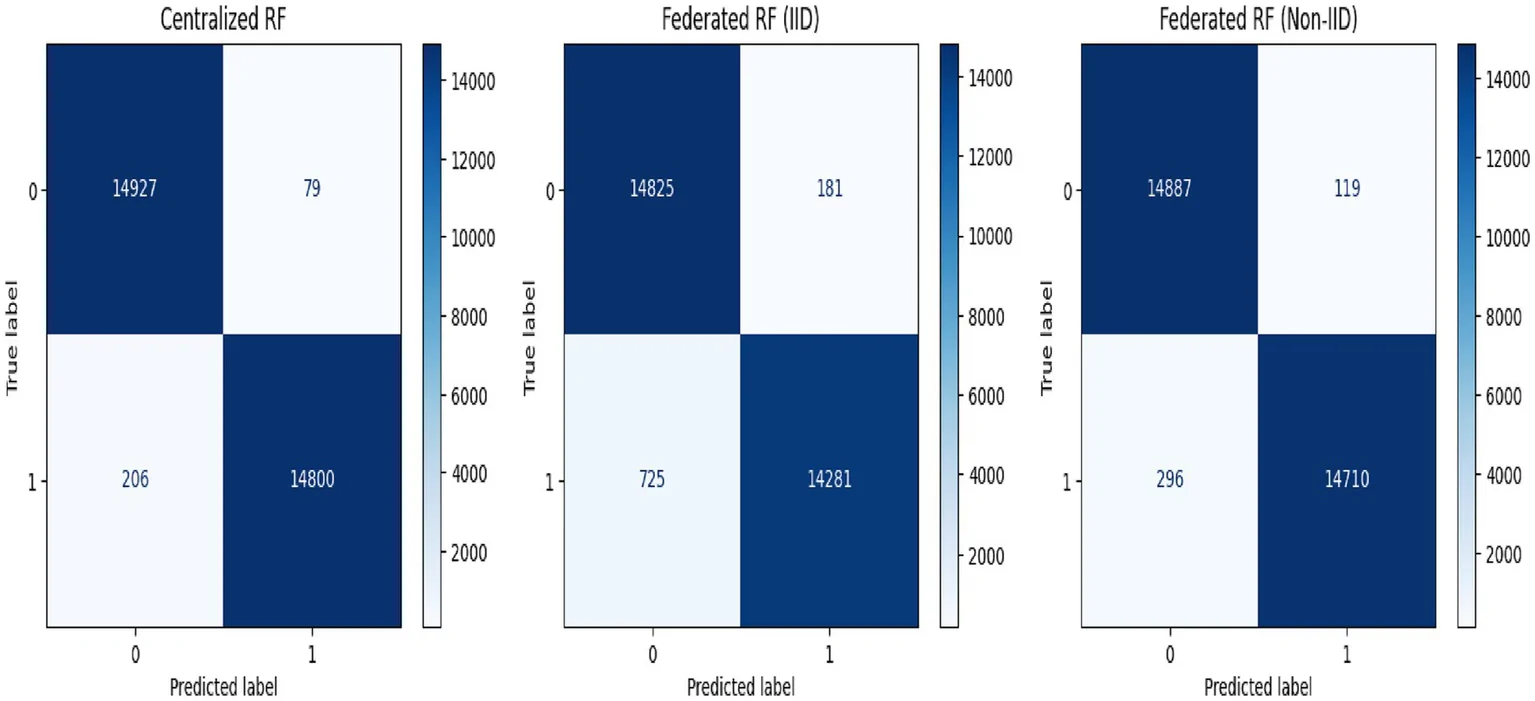
Confusion matrices for centralized and federated models.

As seen in Figure 15, most of the predictions are found to be falling in their right classes. Very low false negatives and false positives are found. This shows that there is high reliability for the proposed system.

**Figure 16:**
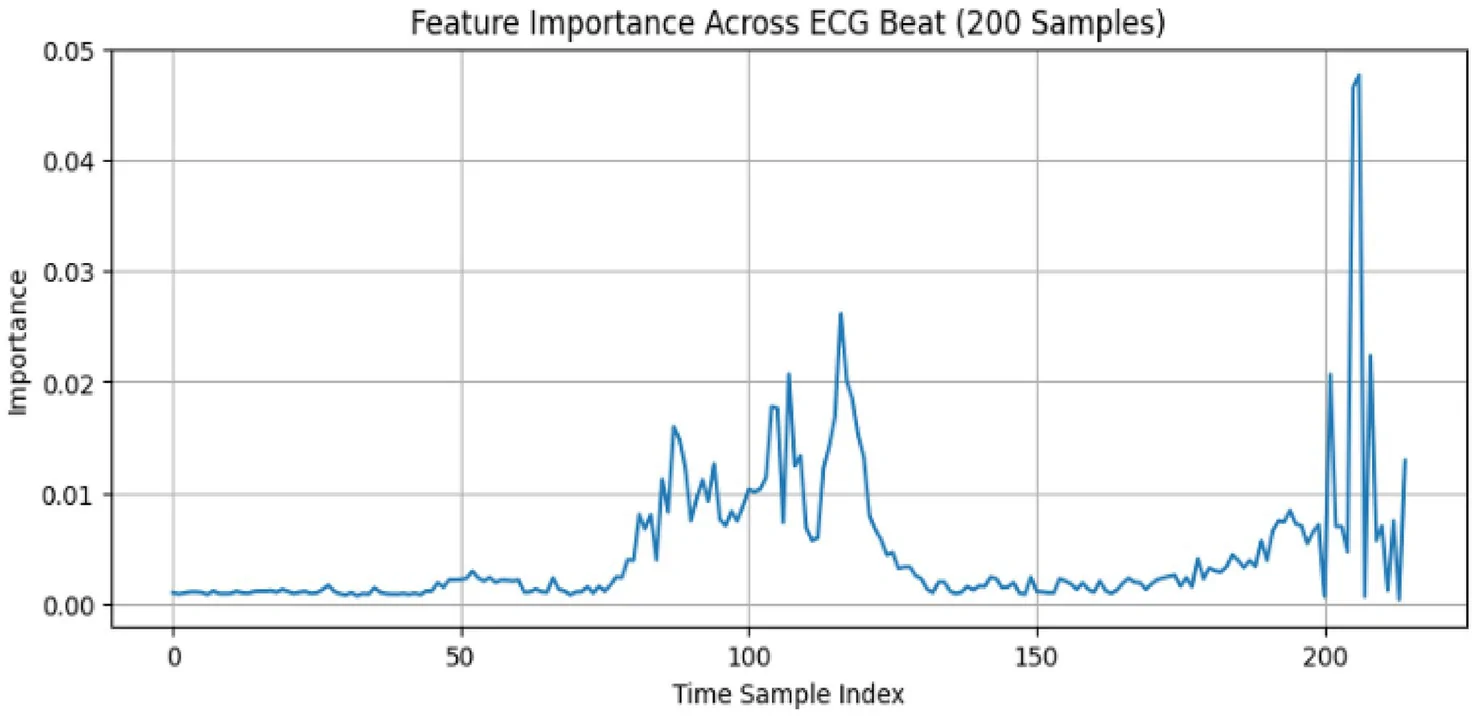
Feature importance across ECG beat samples.

Figure 16 shows the contribution of various time samples within an ECG beat record for classification. This shows that there are certain regions in the signal that show higher importance values. There is a strong influence of these features in differentiating between normal and arrhythmic patterns. Feature extraction and fusion process are effective and plays a major role in capturing the critical characteristics of the ECG signals.

## 8 Conclusion

This proposed work focuses on building a semi-supervised federated learning framework for ECG-based detection of arrhythmia. The system works by integrating feature extraction, adaptive feature fusion, semi-supervised learning and federated learning in order to build an efficient and privacy preserving classification model. The experiments show even though the centralized model achieve the good accuracy as it has complete access to the dataset, the semi-supervised federated learning method achieves an accuracy of 96.81%. The federated setup avoids direct sharing of raw ECG data among the simulated clients, which may reduce direct data-exposure risks compared with centralized raw-data sharing. The non-IID federated setup also maintains competitive performance under the simulated heterogeneous data distribution and achieves an accuracy close to that of the centralized model. Effective utilization of unlabeled data is incorporated using semi-supervised learning by using pseudo labeling. This helps in significantly increasing the number of data used for training without the need for manual annotation. High confidence predictions are only included by using adaptive threshold mechanism. This helps maintain model reliability. The features extracted; raw, statistical and wavelet features are combined using adaptive feature fusion mechanism. This helps in enhancing the model’s ability to identify meaningful patterns in the ECG. High AUC score of 0.9957 and consistent performance was also achieved while performing federated rounds. This shows that there is effectiveness and stability of the proposed system. Overall, the proposed system demonstrates a balance between classification performance, data utilization and distributed training under the simulated federated setting. The present experiment shows the feasibility of the proposed system under the simulated federated setting. However, the study is again limited to one single database, simulated federated clients and binary classification. Hence, the findings made cannot yet establish the real world scalability, clinical applicability or generalizability across different patient populations and healthcare environments. Further validation on external datasets and real world federated environments is required. As future works, the system can be tested using other deep learning models and transformer architectures also. Real time deployment of the model can be taken into consideration by integrating the model with wearable ECG monitoring devices. Advanced federated learning techniques can be employed like secure aggregation and differential privacy. Large and more diverse ECG datasets can be used. Communication overhead in federated learning environments can be reduced by using optimization techniques. Focus can be given on developing communication-efficient aggregation strategies and adaptive federated optimization techniques so that it can further enhance the scalability and robustness of the proposed framework in real-world clinical environments. Furthermore, additional heartbeat categories and appropriate annotation mapping can be incorporated in the same semi-supervised federated learning framework to distinguish among multiple arrhythmia types. This focus on multi-class ECG datasets could help further enhance the clinical applicability.

## Data Availability

The raw data supporting the conclusions of this article will be made available by the authors, without undue reservation.
